# Fluorescent protein-based Zn^2+^ sensors reveal distinct responses of aerobic and anaerobic *Escherichia coli* cultures to excess Zn^2+^

**DOI:** 10.1016/j.jbc.2024.107840

**Published:** 2024-09-30

**Authors:** Hazel N. Nguyen, Uyen Huynh, Melissa L. Zastrow

**Affiliations:** Department of Chemistry, University of Houston, Houston, Texas, United States

**Keywords:** zinc, metal, *Escherichia coli*, biosensor, fluorescence, FRET, microscopic imaging, protein chemistry

## Abstract

Zinc ions are required by all known organisms. Maintaining zinc homeostasis by preventing toxic overload while ensuring sufficient acquisition for cellular functions is crucial for survival and growth of bacteria. Bacteria, however, frequently encounter and must survive in various environments. During infection in host animals, for example, bacteria are exposed to acidic conditions in the stomach and anaerobic conditions in the intestines, but the effects of oxygen on zinc homeostasis in *Escherichia coli* have not been well-studied. Previously, we reported a flavin-binding fluorescent protein-based zinc sensor, CreiLOV_N41C_, which can respond to changes in labile Zn^2+^ levels in bacteria under both aerobic and anaerobic conditions. Here, we combined the use of CreiLOV_N41C_ with established oxygen-dependent fluorescent protein-based sensors, inductively coupled plasma-mass spectrometry, and growth curves to evaluate how oxygen levels affect zinc uptake in *E. coli*. Inductively coupled plasma-mass spectrometry results showed that cells grown aerobically with added zinc acquired more zinc, but no additional zinc was accumulated when cells were grown anaerobically. Using oxygen-independent CreiLOV_N41C_ and the oxygen-dependent ZapCY series of sensors, intracellular labile zinc was detected in *E. coli* grown with varied zinc under varied conditions. Although little to no endogenous zinc was detected by any sensor in *E. coli* cells grown with up to 2 mM added zinc, CreiLOV_N41C_ revealed that when Zn^2+^ was added and detected by cells in real-time, anaerobic cells required more Zn^2+^ to similarly saturate the sensor. Overall, this work reveals that zinc uptake in *E. coli* is impacted by oxygen levels during cell growth.

Zinc is an essential trace element for all living organisms and plays crucial roles in numerous cellular processes. Zinc ions serve as biological signaling molecules, catalytic cofactors, and are important for structural stabilization of many proteins (1, 2, 3). The total cellular zinc content, or zinc quota, varies from ∼10^8^ atoms/mammalian cell to ∼10^7^ atoms/yeast cell and ∼10^5^ atoms/bacterial cell, but when adjusted for the different cell volumes, the total cellular zinc concentration is in the range of 0.1 to 0.5 mM (4, 5, 6, 7, 8). Intracellular zinc can be divided into a large pool of inaccessible zinc ions which are usually tightly bound to proteins and a smaller pool of labile zinc ions that are accessible or loosely bound to small molecules or proteins. To maintain cellular zinc ions at adequate levels to fulfill the nutrient requirement while excluding toxic excess, cells use a variety of transport, storage, and chaperone proteins (9, 10, 11). Although zinc is required for many cellular functions, protein mismetallation can occur in the presence of excess Zn^2+^ ions, thereby disrupting cellular functions and inhibiting bacterial cell growth (12, 13). Bacteria in the gastrointestinal tract of a host organism must compete for Zn^2+^ ions. As an important component of nutritional immunity, host organisms can restrict zinc bioavailability or impose a toxic overload of zinc on bacterial pathogens (14, 15, 16, 17). The mechanisms involved in maintaining zinc homeostasis have been relatively well-studied for model organisms like *Escherichia coli* and various pathogens, but many studies have focused on changes directly imposed by varied metal availabilities (17, 18, 19). Bacteria in different growth environments can encounter numerous environmental changes, including in oxygen levels, which are low in the gastrointestinal tract (20). *E. coli* is known to undergo various metabolic changes in the transition from aerobic to anaerobic growth, and changes in copper and iron uptake have been examined under these conditions, but the impact of oxygen levels on zinc homeostasis is not understood (21, 22, 23, 24, 25).

One approach to studying the mechanisms involved in cellular metal uptake, transport, and storage is to use fluorescent metal ion sensors. Many fluorescent Zn^2+^ sensors have been developed over the last few decades, ranging from synthetic small molecule probes to genetically encoded sensors and chemigenetic probes (26, 27, 28). Genetically encoded fluorescent protein-based sensors are particularly attractive for detecting biological zinc because they can be selectively expressed in specific cell types, tissues, or organisms. For bacteria, which are typically found in multispecies ecosystems, this feature is especially beneficial. Fluorescent protein-based sensors have been broadly used to detect, quantify, and track labile pools of zinc ions, but have thus far been applied primarily to mammalian systems (28). The small size and crowded intracellular environment of bacterial cells requires sensors to be expressed at low enough levels to avoid aggregation while maintaining sufficient brightness and sensitivity for metal detection. Additionally, many bacteria, including a large fraction of gut bacteria, are anaerobes and cannot grow in the presence of oxygen. Most fluorescent protein-based zinc sensors to date are based on the green fluorescent protein (GFP), which requires oxygen for maturation of the fluorescent chromophore (29, 30).

Our group recently reported the first oxygen-independent fluorescent protein-based zinc ion sensor using a mutant of a flavin-binding fluorescent protein, CreiLOV (31). CreiLOV noncovalently binds the ubiquitous flavin mononucleotide cofactor and does not require oxygen for fluorescence (32). CreiLOV has an inherent sensitivity to copper(I) and copper(II) ions and can be quenched with dissociation constants of 12 μM and 0.1 nM, respectively (31, 33). By introducing an additional cysteine in the hypothesized copper binding site, we generated CreiLOV_N41C_, which simultaneously exhibited an improved copper affinity and a new zinc sensitivity (31). Zinc ions quench the fluorescence of CreiLOV_N41C_ reversibly and with *K*_d_ = 1 nM. CreiLOV_N41C_ exhibits strong binding affinity to Cu^2+^ (*K*_d_ = 0.066 fM). Using *E. coli* BL21(DE3) cells, we showed that CreiLOV_N41C_ could respond reversibly to exogenously added zinc ions and could also be used to monitor changes in endogenous zinc when cells were grown in media with varied concentrations of zinc. Since *E. coli* BL21(DE3) cells do not grow well anaerobically, we tested the function of the sensor under anaerobic conditions using *E. coli* MG1655 cells. We found that different concentrations of zinc in the growth medium were required to elicit a sensor response in aerobic *versus* anaerobic conditions. CreiLOV_N41C_ was noticeably quenched by endogenous zinc in aerobically grown *E. coli* when the medium contained as little as ∼0.5 to 1.0 mM ZnCl_2_. On the other hand, at least 2.0 mM ZnCl_2_ was required to elicit a response in anaerobically grown *E. coli* MG1655. At the time, it was not clear if this difference was due to the different oxygen levels or the different *E. coli* strains.

In this work, we aimed to use CreiLOV_N41C_ to monitor zinc dynamics using the same strain of *E. coli* (MG1655). We also evaluated the effects of varied zinc concentrations on *E. coli* growth under aerobic and anaerobic conditions by analyzing the growth kinetics, comparing the effects of different zinc salts, and quantifying total metal ion uptake using inductively coupled plasma-mass spectrometry (ICP-MS). At the same time, we sought to compare the performance of CreiLOV_N41C_ in aerobically grown *E. coli* with a well-established GFP-based zinc sensor, ZapCY2 (34). ZapCY2 is a Förster resonance energy transfer (FRET)-based sensor where the donor protein, cyan fluorescent protein, is connected to the acceptor, yellow fluorescent protein, by a zinc-finger based zinc binding domain. Zinc binding modulates the FRET response and with sensor calibration and prior knowledge of the zinc dissociation constant, ZapCY2 can be used to quantify Zn^2+^ ions. Given that CreiLOV_N41C_ and ZapCY2 have similar affinities for Zn^2+^ ions (*K*_d_ = 0.8–1.0 nM), we reasoned that the sensors should yield similar results when monitoring zinc in aerobically grown *E. coli* (31, 34). Collectively, we found that the total zinc content of aerobically grown *E. coli* increased when Zn^2+^ was added to the medium but stayed the same for anaerobically grown *E. coli*. The growth kinetics of anaerobically grown *E. coli* were impaired with excess Zn^2+^ regardless of the ion source (organic gluconate or inorganic chloride), but Zn^2+^-gluconate was less toxic to aerobically grown *E. coli* at high levels. All fluorescent protein-based sensors tested, oxygen-independent CreiLOV_N41C_ and oxygen-dependent ZapCY1-2, responded to exogenously added zinc in aerobically grown *E. coli.* Neither CreiLOV_N41C_ nor ZapCY2 detected endogenous zinc in *E. coli* grown aerobically in 2 mM ZnCl_2_, but the higher affinity ZapCY1 sensor (*K*_d_ = 2.53 pM) did (34). When Zn^2+^ influx was measured in real-time, both CreiLOV_N41C_ and ZapCY2 responded to Zn^2+^ in aerobically grown *E. coli*. We found that higher concentrations of Zn^2+^ were required to detect real-time Zn^2+^ influx in anaerobically grown cells, confirming that the oxygen level directly impacts zinc uptake. Overall, by using complementary zinc detection methods, ICP-MS for total cellular zinc and fluorescent sensors for labile zinc, we demonstrated that the oxygen level in the growth environment alters zinc homeostasis in *E. coli*. Moreover, CreiLOV_N41C_ was shown to be a powerful tool for tracking zinc in aerobic and anaerobic live *E. coli* cells.

## Results

### Zn^2+^ effects on growth of *E. coli* under aerobic and anaerobic conditions

**Figure 1:**
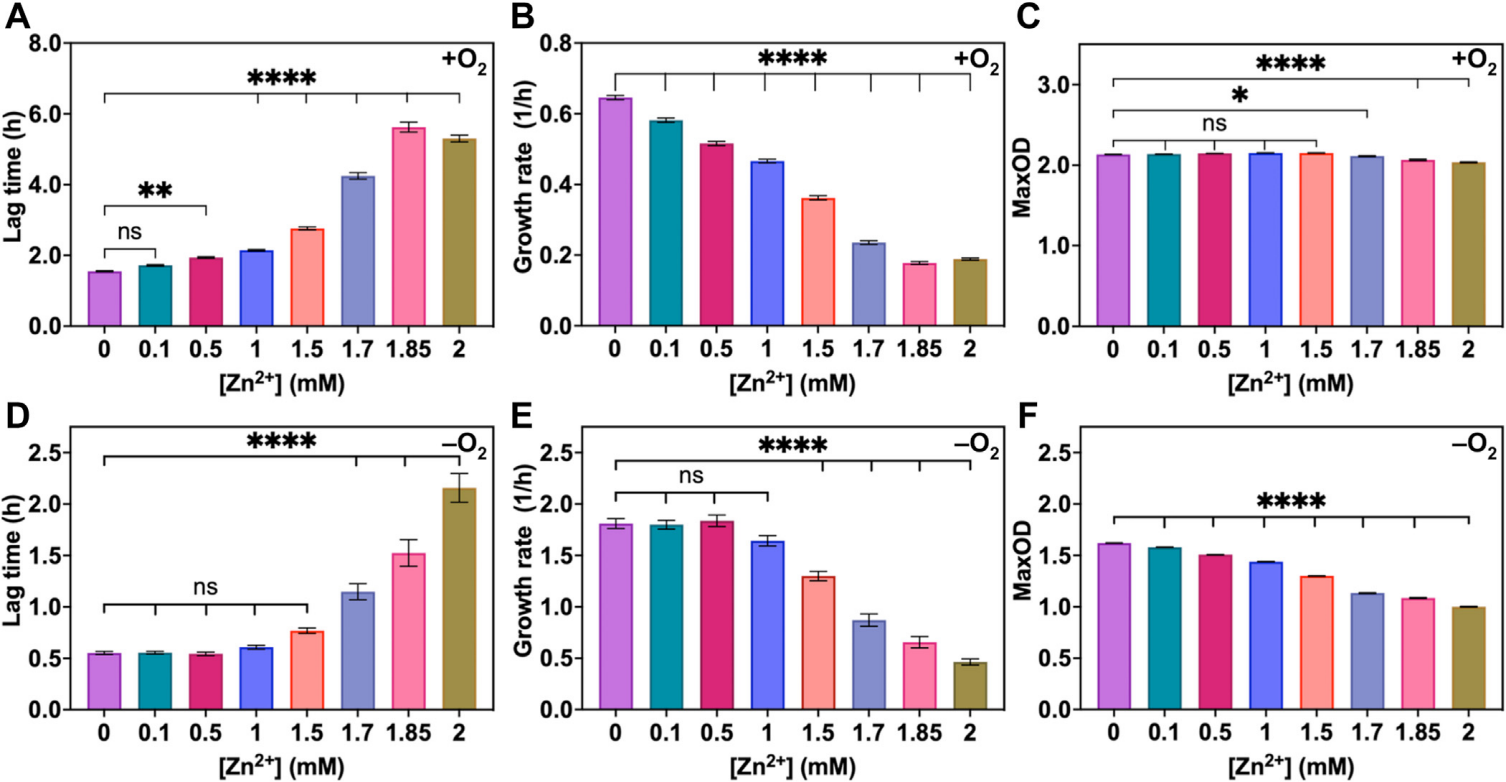
**Effects of varied ZnCl**_**2**_**concentrations on growth kinetics parameters for *Escherichia coli* grown under aerobic (labeled with +O**_**2**_**) and anaerobic (labeled with –O**_**2**_**) conditions**. *A*–*C*, growth parameters for aerobically grown *E. coli*, (*D*–*F*) growth parameters for anaerobically grown *E. coli.* Lag time, growth rate, and max OD_600_ represent the mean ± SEM of three biological replicates for each condition and Zn^2+^ concentration. Growth parameters were calculated from growth curves (Fig. S1, *A* and *B*) using nonlinear regression curve fitting to a four-parameter Logistic equation with GraphPad Prism 9 software. NS, not significant; *∗p* ≤ 0.05; *∗∗p* ≤ 0.01; ∗*∗∗∗p* ≤ 0.0001 as determined by one-way ANOVA with Tukey multiple comparison test.

We investigated the effects of Zn^2+^ on the growth kinetics of *E. coli* cultured under aerobic and anaerobic conditions. For both aerobic and anaerobic conditions, *E. coli* was grown in LB, a rich medium, supplemented with ZnCl_2_ (0–2 mM), and the OD_600_ was recorded until the cells reached the stationary phase (Fig. S1, *A* and *B*). The resulting growth curves were each fitted for the growth parameters, lag time, growth rate, and the maximum optical density at 600 nm (max OD, Fig. 1). As the concentration of added Zn^2+^ increased, the lag time for *E. coli* grown aerobically increased and the growth rate decreased (Fig. 1, *A* and *B*). The presence of higher Zn^2+^ concentrations in the growth medium, however, did not affect the maximum OD_600_ (Fig. 1*C*). Previously, we found no effects of Zn^2+^ on another strain of *E. coli*, BW25113, when grown aerobically with Zn^2+^ up to 1 mM in minimal medium (35). When *E. coli* was grown anaerobically, the lag time and growth rate followed the same trends as observed for aerobic growth conditions; however, the maximum OD_600_ of anaerobic *E. coli* was negatively affected by increased Zn^2+^ concentrations (Fig. 1, *D*–*F*). These results suggest that high Zn^2+^ concentrations (>∼1 mM) are detrimental to both aerobically and anaerobically grown *E. coli.* Additionally, anaerobically grown *E. coli* is more susceptible to Zn^2+^ toxicity since the max OD level decreased with increasing Zn^2+^ concentrations, whereas aerobic *E. coli* grew slower with added Zn^2+^ but was able to recover and reach a similar max OD for all Zn^2+^ concentrations.

Given that organic zinc sources may have higher zinc bioavailability and different impacts on bacterial growth than inorganic zinc sources (36, 37), we also investigated whether zinc gluconate, an organic zinc source, would have similar effects on the growth of *E. coli* as ZnCl_2_, an inorganic zinc source. When *E. coli* was grown aerobically in LB medium containing varied concentrations of zinc gluconate, high Zn^2+^ concentrations had minor effects on lag time and growth rate and no effect on the maximum OD_600_ (Figs. S1*C* and S2, *A*–*C*). This result suggests that aerobically grown *E. coli* is more tolerant to high concentrations of zinc gluconate compared to ZnCl_2_. When *E. coli* was grown anaerobically in LB with 1.5 to 2 mM Zn gluconate, the lag time increased while the growth rate and maximum OD_600_ decreased (Figs. S1*D* and S2, *D*–*F*). Regardless of the zinc source, the growth of *E. coli* under anaerobic conditions was significantly reduced by high Zn^2+^ concentrations. We also grew *E. coli* under aerobic conditions with varied concentrations of ZnSO_4_, another inorganic zinc source, and compared the results to *E. coli* grown with ZnCl_2_ and zinc gluconate. The trend for *E. coli* grown with added ZnSO_4_ was the same as for *E. coli* grown with added ZnCl_2_ (Fig. S3). Collectively, the inorganic zinc sources have a greater impact on the aerobic growth of *E. coli* compared to the organic zinc source, but the inorganic and organic zinc sources have similar effects on the anaerobic growth of *E. coli.* Given that similar max OD_600_ values of *E. coli* were reached for both added ZnCl_2_ and Zn-gluconate sources, particularly when grown aerobically, ZnCl_2_ was used for the remaining experiments.

### Quantification of the total zinc content of *E. coli* grown aerobically and anaerobically

**Figure 2:**
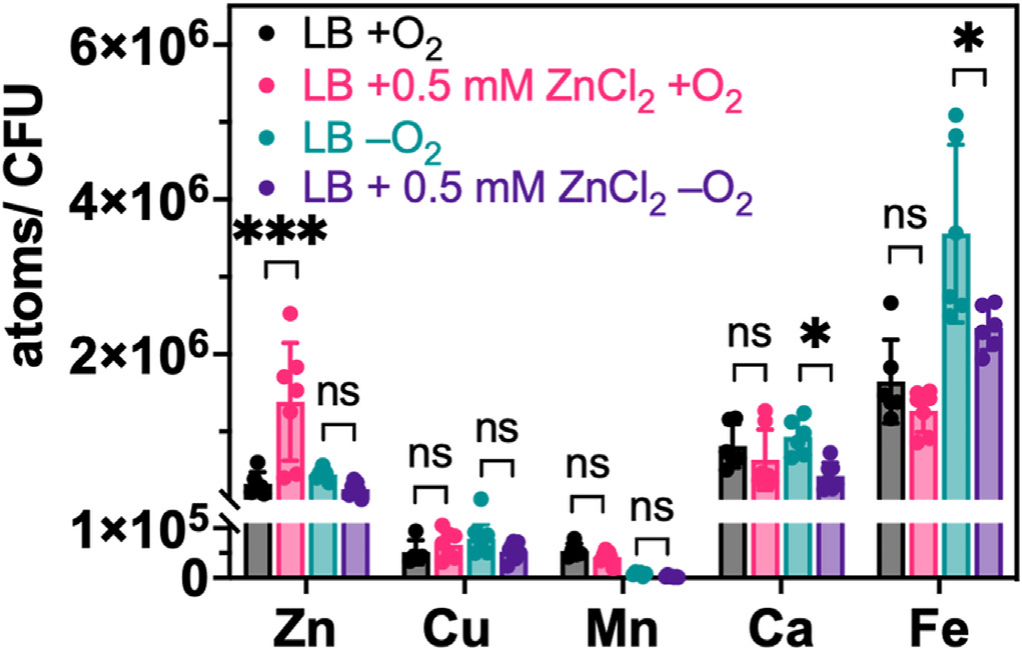
**ICP-MS quantification of metal contents of *Escherichia coli* grown aerobically (+O**_**2**_**) and anaerobically (–O**_**2**_**) in LB and LB containing 0.5 mM added ZnCl**_**2**_. Data are shown as individual data points overlaid on the bar representation, with error bars as SD from three biological replicates. NS, not significant; *∗p* ≤ 0.05; *∗∗∗p* ≤ 0.001 as determined by one-way ANOVA with Tukey multiple comparison test.

Because *E. coli* grew more slowly with high concentrations of Zn^2+^ under aerobic and anaerobic conditions and reached a lower culture density (max OD_600_) under anaerobic conditions, we used ICP-MS to determine whether the total cellular zinc content would similarly vary with the growth conditions. Here, we grew *E. coli* with 0.5 mM ZnCl_2_ because this is the highest concentration tested that had minimal effects on the growth kinetics of *E. coli* (Fig. 1). *E. coli* was grown in LB and LB supplemented with 0.5 mM ZnCl_2_ under aerobic and anaerobic conditions until the culture densities reached the exponential phase (OD_600_ = 0.6–0.8). The cells were harvested, washed, and digested with trace metal grade concentrated HNO_3_ prior to ICP-MS measurements. The metal concentrations in the media were analyzed by inductively coupled plasma-optical emission spectrometry (ICP-OES). For LB and LB containing 0.5 mM ZnCl_2_, the measured zinc concentrations are 10 and ∼490 μM, respectively (Table S1 and Fig. S4). For cells grown under aerobic conditions, the total zinc content measured was increased by over 4-fold with 0.5 mM added ZnCl_2_, suggesting that *E. coli* can acquire more zinc when the zinc concentration increases (Fig. 2). Surprisingly, when *E. coli* was grown anaerobically in medium containing 0.5 mM ZnCl_2_, the cells did not accumulate additional zinc compared to when cells were grown anaerobically in medium without added zinc (Fig. 2). Based on the growth kinetics of *E. coli* and this ICP-MS analysis, we can suggest that the lag time and growth rate of *E. coli* grown aerobically in 0.5 mM ZnCl_2_ were moderately impacted due to increased zinc accumulation, whereas the lag time and growth rate of anaerobic *E. coli* were unaffected at least in part because the cells did not take up the excess zinc from the medium.

We also measured the total metal contents in *E. coli* for several other metals (Figs. 2 and S5). For most biologically relevant metals, there were no significant changes in accumulation of the metal with supplementation of zinc, especially under aerobic conditions (Figs. 2, S5*A*, and Table S2). Under anaerobic conditions, there were some effects of oxygen and zinc supplementation on metal uptake (Figs. 2 and S5*B*, and Table S3). Among the abundant main group elements, calcium and potassium accumulation decreased by ∼2.2-fold with added zinc when *E. coli* was grown under anaerobic conditions. By comparison, the accumulation of these metals with added zinc under aerobic conditions did not change significantly. Selenium and molybdenum contents also decreased by a similar amount under anaerobic conditions, although these metals are significantly less abundant. Among the first-row transition metals, accumulations of iron and nickel were also reduced under anaerobic growth conditions, by ∼1.5-fold for iron and almost 3-fold for nickel. Pairwise comparisons of metal accumulation for cells grown in the same medium conditions but aerobically or anaerobically reveal additional differences in accumulation (Fig. S5, *C* and *D*). For example, the supplementation of zinc does not affect manganese contents, but when comparing anaerobically grown cells to aerobically grown cells, ∼7-17-fold less manganese was detected in anaerobic cells. On the other hand, several metals showed significant increases in accumulation under anaerobic conditions, including magnesium, iron, and molybdenum. *E. coli* has been previously reported to acquire more copper under anaerobic conditions, and although we did not detect significant changes in this work, there is a slight increase in copper for cells grown in LB and an even smaller decrease for cells grown in LB with supplemented ZnCl_2_ (21). Overall, these results demonstrate distinct patterns for the effects of supplemented zinc and aerobic *versus* anaerobic growth on cellular metal uptake.

### Detecting intracellular labile Zn^2+^ in *E. coli*

**Figure 3:**
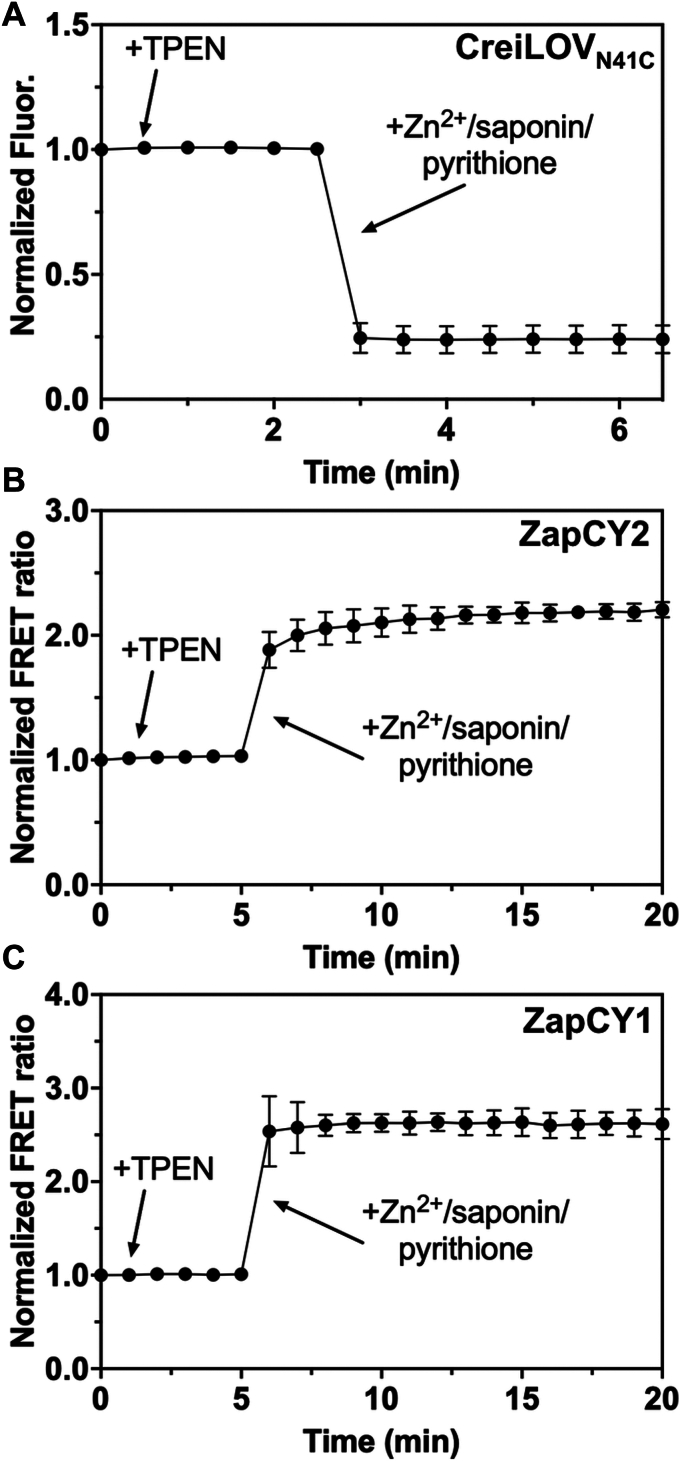
**Intracellular zinc in aerobic live *Escherichia coli* cells detected by fluorescent protein based Zn**^**2+**^**sensors**. (*A*) CreiLOV_N41C_, (*B*) ZapCY2, and (*C*) ZapCY1. The fluorescence emission of CreiLOV_N41C_ was recorded initially and every 30 s after addition of TPEN (50 μM) and ZnCl_2_/saponin/pyrithione (100 μM/0.002%/1.5 μM). The fluorescence emission of ZapCY2 and ZapCY1 was recorded initially and every 1 min after addition of TPEN (5 μM) and ZnCl_2_/saponin/pyrithione (10 μM/0.002%/1.5 μM). Buffer: 50 mM HEPES, 100 mM NaCl, pH 7.1. λ_ex_ = 450 nm (CreiLOV_N41C_) and 433 nm (ZapCY2 and ZapCY1). All error bars represent the standard deviation for three biological replicates. TPEN, *N,N,N′,N′*-tetrakis-(2-pyridylmethyl)-ethylenediamine. HEPES, 4-(2-Hydroxyethyl)piperazine-1-ethanesulfonic acid.

The zinc quota is distributed into a large pool of zinc ions that are tightly bound to proteins and a smaller pool of labile zinc ions. To better understand the changes in zinc uptake in *E. coli* grown aerobically and anaerobically, we measured the intracellular labile zinc using fluorescent protein-based zinc sensors. Previously, we showed that CreiLOV_N41C_ is an excellent oxygen-independent zinc reporter for bacteria grown under either aerobic or anaerobic conditions (31). Here, we applied CreiLOV_N41C_, which has a nanomolar dissociation constant for Zn^2+^, in *E. coli* to detect changes in the labile zinc pool. For comparison, we used two GFP-based Zn^2+^ sensors, ZapCY2 (*K*_d_ = 811 pM) and ZapCY1 (*K*_d_ = 2.5 pM), which were previously applied to detect zinc in mammalian cells, but not in *E. coli* (34, 38). Initially, CreiLOV_N41C_ was aerobically expressed using the same growth and protein expression conditions (0.5 mM IPTG, 37 °C, 3 h) as previously reported, but fluorescence microscopy analyses revealed an uneven distribution of fluorescence in the cells, possibly due to the formation of inclusion bodies (Fig. S6*A*) (31). After optimizing the protein expression conditions (0.1 mM IPTG, 18 °C, overnight), no inclusion bodies were observed (Fig. S6*B*). The ZapCY series sensors were also expressed in *E. coli* grown at 18 °C overnight. Using a live cell suspension expressing each of the sensors, labile Zn^2+^ was detected by first measuring the initial fluorescence intensity, then measuring the fluorescence after the addition of *N,N,N′,N′*-tetrakis-(2-pyridylmethyl)-ethylenediamine (TPEN) and, subsequently, with the addition of ZnCl_2_/saponin/pyrithione (Figs. 3 and S7). For CreiLOV_N41C_, the results shown are based on changes in the fluorescence intensity whereas for the ZapCY series, the changes in the FRET ratio are shown. The FRET ratio is calculated by dividing the fluorescence intensity of the acceptor (yellow fluorescent protein) by the fluorescence intensity of the donor (cyan fluorescent protein). There was no fluorescence enhancement of CreiLOV_N41C_ after the addition of TPEN, indicating no detectable endogenous zinc in *E. coli* (Figs. 3*A* and S7*A*). Subsequent addition of ZnCl_2_/saponin/pyrithione quenched the fluorescence of CreiLOV_N41C_, suggesting that *E. coli* took up a high amount of exogenous zinc. This is consistent with previously reported results using CreiLOV_N41C_ in *E. coli* MG1655 cells grown anaerobically (31). Similarly, ZapCY2 was used to detect zinc in live aerobically grown *E. coli* cells, and there was no change in the FRET ratio after the treatment of TPEN and an increase in FRET ratio upon the addition of ZnCl_2_/saponin/pyrithione (Figs. 3*B* and S7*B*). The intracellular physiological concentration of zinc in *E. coli* is thought to be lower (∼10^−15^–10^−12^ M) than in many mammalian cells (∼10^−10^–10^−9^ M) (27, 39, 40, 41). Given that CreiLOV_N41C_ and ZapCY2 have dissociation constants in the nanomolar range (1 nM and 0.811 nM, respectively), we hypothesized that any endogenous Zn^2+^ would likely not be within the detection limit range of the probes (31, 34). We repeated the above experiment using ZapCY1, which has a stronger binding affinity to zinc (*K*_d_ ≈ pM) (34, 38). When treated with TPEN, the FRET ratio of ZapCY1 did not decrease (Figs. 3*C* and S7*C*). ZapCY1 also detected no endogenous Zn^2+^ in *E. coli*, consistent with a physiological zinc concentration closer to femtomolar than picomolar levels in these bacteria. The FRET ratio of ZapCY1 increased upon saturation with exogenous Zn^2+^. These data show that *E. coli* accumulated no detectable endogenous labile Zn^2+^ under these growth conditions but confirm that these sensors can be used to reversibly monitor exogenously added Zn^2+^ in bacteria. These experiments are reproducible in bacteria that do not form inclusion bodies during protein expression, but in samples where inclusion bodies were formed the sensors can yield inconsistent results.

**Figure 4:**
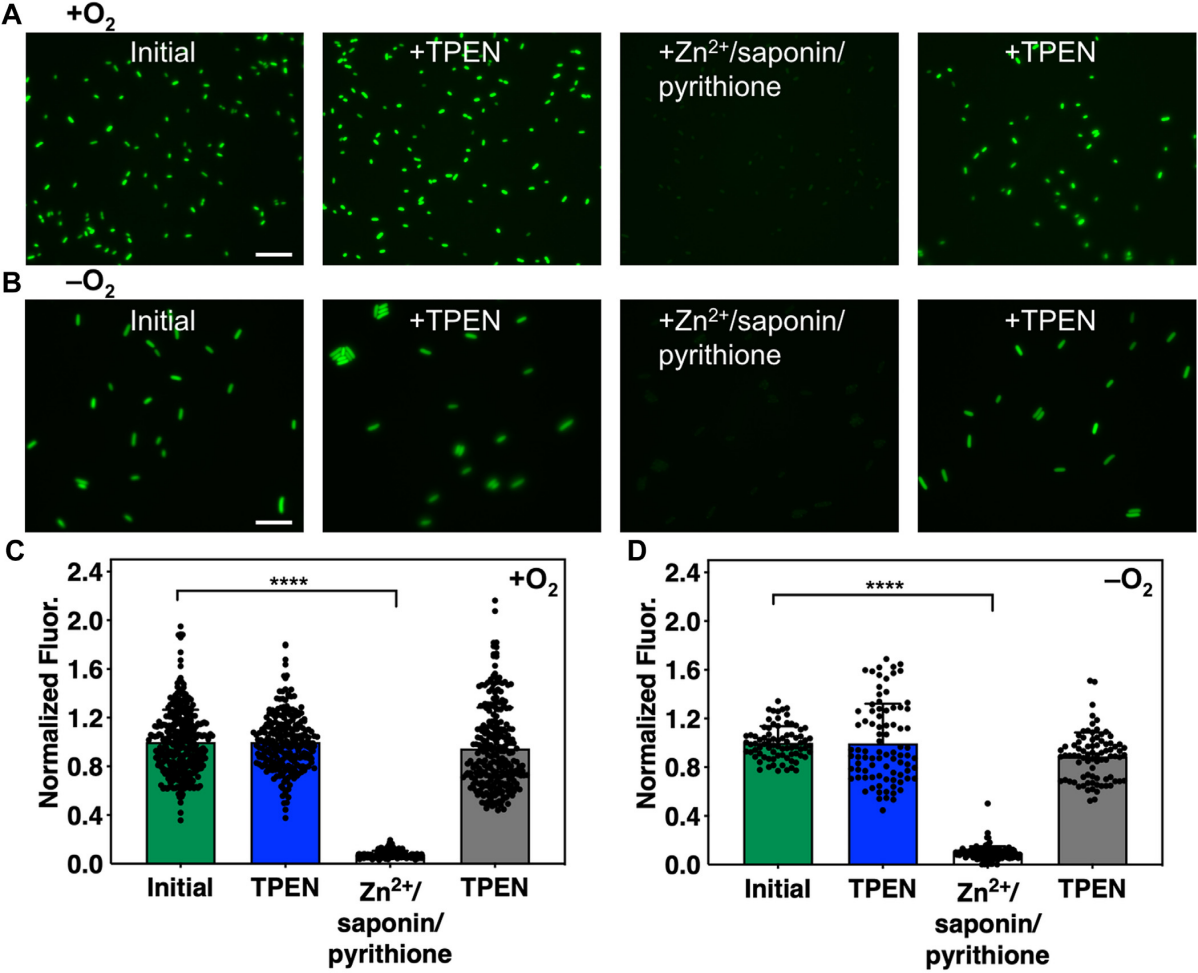
**Fluorescence microscopy of *Escherichia coli* expressing CreiLOV**_**N41C**_**under aerobic and anaerobic conditions.***A*, aerobically grown and imaged *E. coli*. *B*, anaerobically grown and imaged *E. coli.* From *left to right* in *A* and *B*: initial fluorescence signal from CreiLOV_N41C_, fluorescence signal 5 min after the addition of 50 μM TPEN, 15 min after the addition of 100 μM ZnCl_2_/0.002% saponin/1.5 μM pyrithione, and 20 min after the addition of 200 μM TPEN. The scale bar represents 10 μm. *C* and *D*, quantification of change in fluorescence signals shown in parts *A* and *B*, respectively. Mean cell intensities were normalized to the initial fluorescence intensity. Individual data points representing individual cells are overlaid on the bar chart representation and were collected from three biological replicates, each with two or more technical replicates. Data were analyzed by means of one-way ANOVA with Tukey multiple comparison test. ∗∗∗∗*p* < 0.0001. Error bars represent the standard deviation. TPEN, *N,N,N′,N′*-tetrakis-(2-pyridylmethyl)-ethylenediamine.

Next, we used fluorescence microscopy to visualize intracellular labile zinc in *E. coli* grown under aerobic and anaerobic conditions (Fig. 4). Aerobically grown *E. coli* cells expressing CreiLOV_N41C_ were imaged by first collecting the initial fluorescence in the green channel, followed by the fluorescence after treatment with TPEN. As for the live cell suspension experiment, the addition of TPEN had no effect on the fluorescence signal of CreiLOV_N41C_, confirming that there is no detectable endogenous zinc in these cells (Fig. 4, *A* and *C*). Addition of ZnCl_2_/saponin/pyrithione quenched the fluorescence, and excess TPEN mostly reversed the fluorescence signal of CreiLOV_N41C_, which is also consistent with the live cell suspension experiment results described above. When grown under anaerobic conditions, *E. coli* cells expressing CreiLOV_N41C_ were also imaged using fluorescence microscopy before and after the addition of TPEN and then after the addition of ZnCl_2_/saponin/pyrithione and subsequently, more TPEN. For anaerobic imaging, cells were grown and handled under anaerobic conditions throughout the whole experiment. Microscope slides were sealed with epoxy to maintain anaerobic conditions (details in Experimental procedures) (42). As for the previously reported live cell suspension (31), no endogenous zinc was detected since the fluorescence signal after TPEN addition did not change (Fig. 4, *B* and *D*). The fluorescence signal was quenched with the addition of ZnCl_2_/saponin/pyrithione and subsequently restored with excess TPEN, further confirming that CreiLOV_N41C_ can be used to monitor exogenous zinc in *E. coli* grown and analyzed in both oxygen and oxygen-free environments.

### Detecting changes in endogenous Zn^2+^ in *E. coli* grown aerobically and anaerobically with varied zinc concentrations

**Figure 5:**
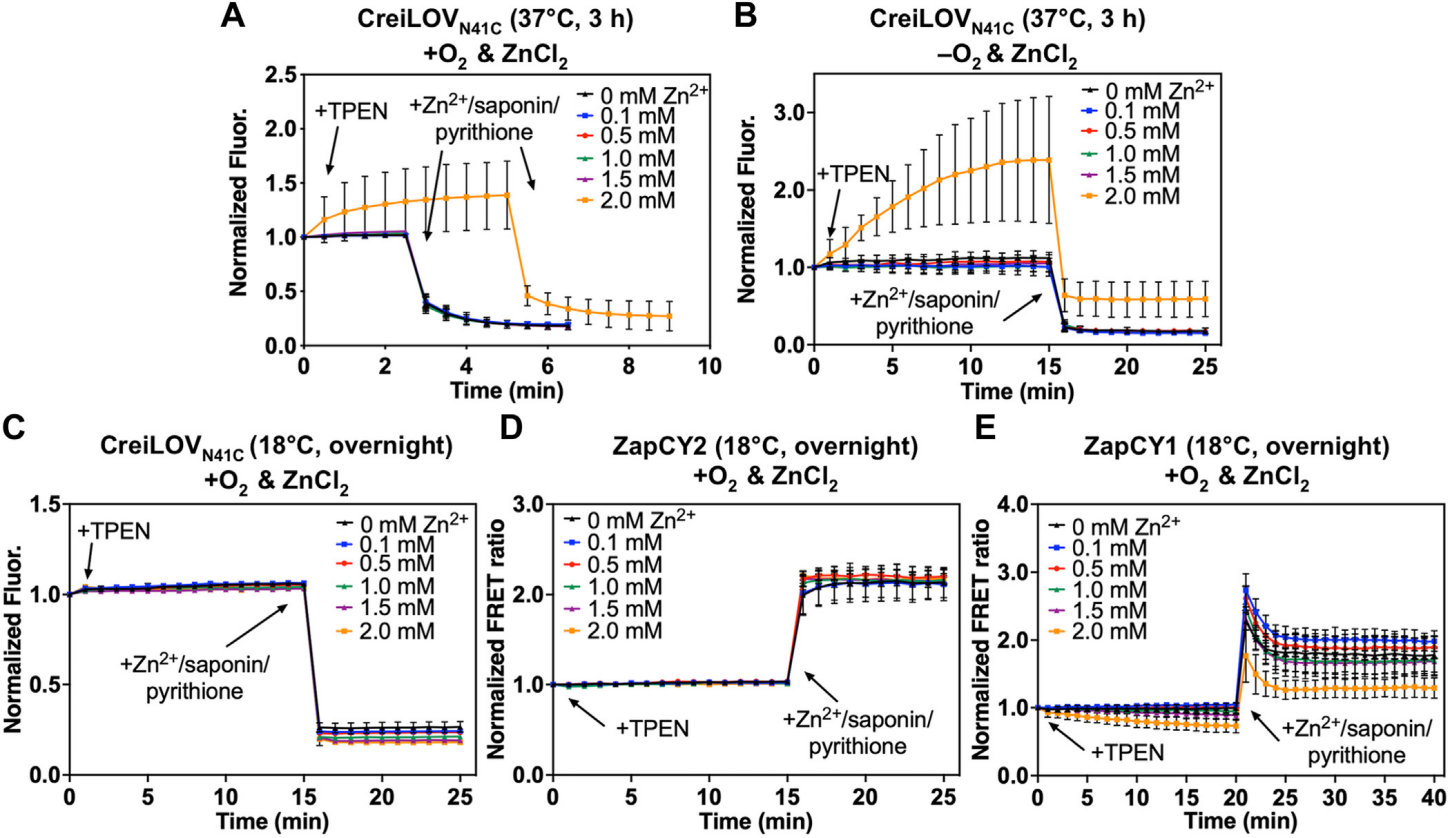
**Endogenous zinc as detected by fluorescent protein-based Zn**^**2+**^**sensors in live cell suspensions of *Escherichia coli* grown aerobically and anaerobically under different protein expression conditions**. Detection of endogenous Zn^2+^ by CreiLOV_N41C_ when protein expression was induced with 0.5 mM IPTG for 3 h at 37 °C and cells were grown (*A*) aerobically or (*B*) anaerobically. Detection of endogenous Zn^2+^ by (*C*) CreiLOV_N41C_, (*D*) ZapCY2, and (*E*) ZapCY1 when protein expression was induced by 0.1 mM IPTG at 18 °C overnight and cells were grown aerobically. Under all conditions, *E. coli* cells were grown in the presence of ZnCl_2_ (0–2 mM). For each sample, the fluorescence emission was recorded initially and every 30 s or 1 min after addition of TPEN (50 μM) and ZnCl_2_/saponin/pyrithione (100 μM/0.002%/1.5 μM). Buffer: 50 mM HEPES, 100 mM NaCl, pH 7.1. λ_ex_ = 450 nm (CreiLOV_N41C_) and 433 nm (ZapCY2 and ZapCY1). All error bars represent the standard deviation for 3 biological replicates, each with ≥2 technical replicates. TPEN, *N,N,N′,N′*-tetrakis-(2-pyridylmethyl)-ethylenediamine. HEPES, 4-(2-Hydroxyethyl)piperazine-1-ethanesulfonic acid.

Our prior work revealed that CreiLOV_N41C_ could detect increased endogenous zinc in *E. coli* MG1655 grown with 2 mM added ZnCl_2_ and slightly increased endogenous zinc in *E. coli* grown with 1.5 mM added ZnCl_2_ under anaerobic conditions (31). Less zinc was required to increase the endogenous Zn^2+^ levels when cells were grown under aerobic conditions in that work, but a different strain of *E. coli* was used, BL21(DE3). Here, we measured the endogenous zinc in aerobically grown *E. coli* MG1655 with varied concentrations of ZnCl_2_ to allow a direct comparison of the effects of oxygen on endogenous Zn^2+^ levels. For these live cell suspension experiments, starter cultures were inoculated in LB medium supplemented with ZnCl_2_ (0–2 mM), and expression of the sensor protein was induced by 0.5 mM IPTG at 37 °C for 3 h. Cells were harvested and transferred to a cuvette for fluorescence analysis. After measuring the initial fluorescence signal, cells were treated with TPEN and the fluorescence was recorded before adding exogenous zinc for signal calibration (ZnCl_2_/saponin/pyrithione). The addition of TPEN after recording the initial signal did not affect the fluorescence of CreiLOV_N41C_ in *E. coli* grown in 0.1 to 1.5 mM ZnCl_2_, but the chelator increased the fluorescence in *E. coli* grown in 2 mM ZnCl_2_, indicating an increased level of endogenous Zn^2+^ in these cells after growth (Fig. 5*A*). This result is different from what we previously observed for a different strain of *E. coli*, BL21(DE3). When grown aerobically, *E. coli* BL21(DE3) cells grown with only ∼0.5 to 1.0 mM added ZnCl_2_ exhibited increased fluorescence upon addition of chelator to the cell suspension, consistent with increased endogenous Zn^2+^. We also repeated the experiment from our prior work for *E. coli* MG1655 grown anaerobically (31), and found increased levels of endogenous Zn^2+^ when cells were grown in medium supplemented with 2 mM ZnCl_2_, but not for cells grown with 1.5 mM supplemented ZnCl_2_ (Fig. 5*B*). This difference could be due to subtle changes in growth of the bacteria or possible formation of inclusion bodies when the protein is expressed at 37 °C with 0.5 mM IPTG (Fig. S6*A*). Therefore, when zinc is included in the growth medium under these conditions, *E. coli* MG1655 cells appear to respond similarly regardless of the presence of oxygen, and endogenous Zn^2+^ is only detected by CreiLOV_N41C_ at the highest added ZnCl_2_ concentration (Fig. 5, *A* and *B*).

Since we observed a slightly different zinc effect on the growth of aerobic *E. coli* when using zinc gluconate as zinc source, we decided to use CreiLOV_N41C_ to monitor endogenous zinc in *E. coli* grown aerobically and anaerobically with zinc gluconate (0–2 mM). The expression of CreiLOV_N41C_ was induced by 0.5 mM IPTG at 37 °C for 3 h. Under both aerobic and anaerobic conditions, there was a minor effect of TPEN on the fluorescence of CreiLOV_N41C_ when *E. coli* was grown in the presence of 2 mM zinc gluconate, whereas no effect was observed when *E. coli* was grown in the presence of 0.1 mM–1.5 mM Zn^2+^ (Fig. S8). These results are similar to those observed for cells grown with varied ZnCl_2_.

As noted above, the initial protein expression conditions (0.5 mM IPTG, 37 °C, 3 h) that we used for expressing CreiLOV_N41C_ for these live cell suspension experiments could result in inclusion bodies (Fig. S6*A*) and impact the reproducibility of these experiments. We therefore optimized the expression conditions to minimize the inclusion bodies (as evaluated by fluorescence microscopy, Fig. S6*B*). The optimized conditions (0.1 mM IPTG, 18 °C, overnight) were used to express CreiLOV_N41C_ under aerobic conditions and analyze the effects of zinc in the growth medium. After TPEN treatment, the fluorescence of CreiLOV_N41C_ expressed in the presence of varied Zn^2+^ concentrations did not increase, signifying that there was no detectable increase in endogenous zinc in *E. coli* grown with ZnCl_2_ (0–2 mM) (Fig. 5*C*). For comparison, we also used the well-established ZapCY2 sensor to monitor endogenous zinc in *E. coli* grown with varied Zn^2+^ concentrations. For all samples, ZapCY2 did not exhibit any changes in the FRET ratio when treated with metal chelator (Fig. 5*D*). No endogenous zinc was detected by ZapCY2 or CreiLOV_N41C_ when *E. coli* was grown in the presence of low to high concentrations of zinc with optimal protein expression conditions. CreiLOV_N41C_ and ZapCY2 both have dissociation constants around 1 nM and could not detect endogenous zinc, suggesting that the zinc concentration is below the nanomolar range. Therefore, we also evaluated endogenous zinc levels in aerobically grown *E. coli* using ZapCY1, which is a higher affinity Zn^2+^ sensor with a *K*_d_ of 2.5 pM (34). ZapCY1 did not detect any increase in endogenous zinc for cells grown with 0.1 mM–1.5 mM added Zn^2+^ (Fig. 5*E*). For cells grown with 2 mM Zn^2+^, however, a small increase in endogenous zinc was evidenced by a reduced FRET ratio with the addition of a chelator. Based on these results, we conclude that *E. coli* accumulates little labile Zn^2+^, up to only ∼3 pM with the inclusion of high ZnCl_2_ (2 mM) when grown under aerobic conditions overnight.

### Sensing Zn^2+^ influx in aerobically and anaerobically grown *E. coli*

**Figure 6:**
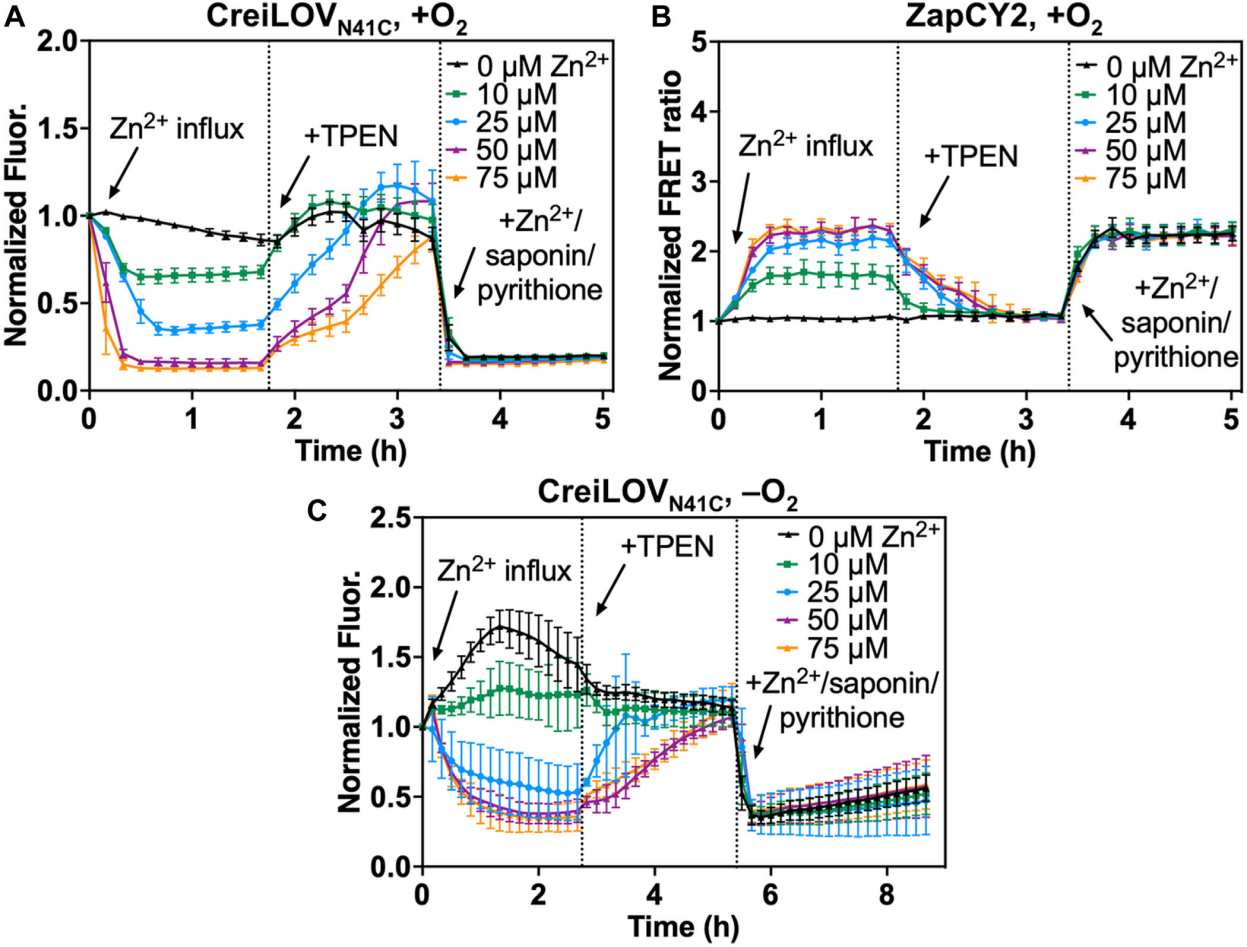
**Zn^2+^ ion influx in live cell suspensions of *Escherichia coli* grown aerobically and anaerobically as monitored by fluorescent protein-based Zn^2+^ sensors.** Zn^2+^ ion influx for *E. coli* grown aerobically (*A* and *B*) and anaerobically (*C*), detected by CreiLOV_N41C_ (*A* and *C*) and ZapCY2 (*B*). For each sample, the fluorescence emission was recorded initially and every 10 min after addition of ZnCl_2_ (0–75 μM), TPEN (100 μM for CreiLOV_N41C_ and 150 μM for ZapCY2) and ZnCl_2_/saponin/pyrithione (200 μM/0.002%/1.5 μM). Buffer: 50 mM HEPES, 100 mM NaCl, pH 7.1. λ_ex_ = 450 nm (CreiLOV_N41C_) and 433 nm (ZapCY2). All error bars represent the standard deviation for three biological replicates, each with ≥2 technical replicates. TPEN, *N,N,N′,N′*-tetrakis-(2-pyridylmethyl)-ethylenediamine. HEPES, 4-(2-Hydroxyethyl)piperazine-1-ethanesulfonic acid.

Since we found that endogenous labile Zn^2+^ levels were too low for detection by CreiLOV_N41C_ and ZapCY2 when *E. coli* cells were grown with varied ZnCl_2_ under optimal protein expression conditions prior to cell harvesting and analysis, we next evaluated whether the sensors could detect Zn^2+^ influx in real time in *E. coli* cell suspensions. *E. coli* was grown in LB and the optimized protein expression conditions (0.1 mM IPTG, 18 °C, overnight) were used for both CreiLOV_N41C_ and ZapCY2. Cells were harvested and transferred to a cuvette containing metal-free 4-(2-Hydroxyethyl)piperazine-1-ethanesulfonic acid (HEPES) buffer. After measuring the initial fluorescence, ZnCl_2_ (0–75 μM) was added to each cell suspension, and the fluorescence was recorded every 10 min for 2 h. For subsequent calibration, TPEN was added, then ZnCl_2_/saponin/pyrithione, and the fluorescence was recorded for each addition. As the concentration of ZnCl_2_ increased from 0 to 75 μM, the quenching of CreiLOV_N41C_ correspondingly increased from 30% for 10 μM ZnCl_2_ up to a maximum of 84% and 87% quenching for 50 μM and 75 μM ZnCl_2_ (Fig. 6*A*). For each addition, the maximum quenching was achieved after 30 min. We note that there was a modest increase in quenching even when no ZnCl_2_ was added to the cells, possibly indicating accumulation of zinc from the buffer for CreiLOV_N41C_-expressing cells (this was not observed for ZapCY2 below). CreiLOV_N41C_ fluorescence was fully restored by excess TPEN for all samples and quenched by excess ZnCl_2_/saponin/pyrithione. Similarly, ZapCY2 detected Zn^2+^ influx with the addition of 10 to 75 μM ZnCl_2_. Normalized FRET ratios of 1.7 and 2.1 were reached with addition of 10 and 25 μM ZnCl_2_, and maximal FRET ratios of 2.3 were achieved with addition of 50 and 75 μM ZnCl_2_ (Fig. 6*B*). Excess TPEN dropped the FRET ratio back to 1 and ZnCl_2_/saponin/pyrithione raised the FRET ratio up to 2.3. Both CreiLOV_N41C_ and ZapCY2 can monitor Zn^2+^ influx in real time in live cell suspensions of *E. coli* grown under aerobic conditions.

Next, we aimed to monitor real time uptake of zinc ions in cells under anaerobic conditions. Here, CreiLOV_N41C_ was expressed at low temperature overnight, and the live cell suspension assay was conducted as described above while maintaining anaerobic conditions. Without added ZnCl_2_, the fluorescence intensity of CreiLOV_N41C_ increased up to 1.7-fold after 80 min and then gradually dropped after 2.7 h (Fig. 6*C*). It is possible that anaerobically grown *E. coli* initially exported excess labile zinc accumulated from the growth medium when transferred to HEPES buffer then later acquired more zinc. ICP-OES analysis reveals that HEPES buffer treated with Chelex-100 contains 0.17 μM Zn^2+^ (Table S4). Although this amount of Zn^2+^ is significantly reduced compared to the amount we found in LB medium (10.5 μM), *E. coli* could acquire some of the zinc remaining in chelexed HEPES buffer. No quenching effect was seen after 10 μM ZnCl_2_ was added to the cell suspension. In the presence of higher concentrations of added ZnCl_2_, zinc influx in anaerobic *E. coli* was observed. We measured 47%, 60%, and 65% quenching of CreiLOV_N41C_ with addition of 25 μM, 50 μM, and 75 μM ZnCl_2_, respectively. The fluorescence intensity quenched by Zn^2+^ could be fully restored with TPEN and excess ZnCl_2_/saponin/pyrithione quenched the fluorescence intensity in all cell suspensions. Overall, CreiLOV_N41C_ can be effectively used to detect zinc influx in *E. coli* under anaerobic conditions.

## Discussion

Fluorescent Zn^2+^ sensors have provided significant insight to the roles of zinc across biology, but few fluorescent protein-based sensors have been applied to study bacterial metal homeostasis and there are few that also function under anaerobic conditions. Here, we demonstrate the utility of a previously reported sensor from our group, CreiLOV_N41C_, which functions independently of oxygen, alongside well-established oxygen-dependent zinc sensors, ZapCY2 and ZapCY1 (31, 34). By combining the use of sensors with bacterial growth curves and ICP-MS analyses to quantify total metal uptake, we show that anaerobically grown *E. coli* MG1655 cells take up less zinc and are more susceptible to toxicity from excess Zn^2+^ in the growth medium.

In our prior work, we showed that CreiLOV_N41C_ could detect exogenous and endogenous zinc when *E. coli* cells were grown aerobically or anaerobically but found that more Zn^2+^ was required during growth to elicit a sensor response in the anaerobic cells (31). We also noticed that the reverse kinetics of the sensor response were slower under anaerobic conditions. Given that two different strains of *E. coli* were used in that work (BL21(DE3) for aerobic analyses and MG1655 for anaerobic analyses), here, we aimed to evaluate the impact of oxygen levels on zinc homeostasis in a single strain of *E. coli*, MG1655. Although the kinetics of the sensor response were similar in this strain regardless of aerobic or anaerobic growth conditions, there were clear differences in zinc uptake, as evidenced by application of the sensors and other methods. Growth kinetics curves revealed similar impacts of varied ZnCl_2_ on aerobic and anaerobic growth parameters of *E. coli*, with the exception of a reduction in the max OD_600_ for anaerobically grown cells as the zinc concentration increased (Figs. 1 and S1, *A* and *B*). Both conditions showed reduced growth rates and increased lag times, but aerobically grown cells recovered to similar max OD_600_ levels up to the highest Zn^2+^ concentration tested, 2 mM. Together, these growth curve analyses suggest that while the growth of *E. coli* is delayed under both aerobic and anaerobic conditions with excess Zn^2+^, aerobically grown *E. coli* were more tolerant to excess Zn^2+^ than anaerobically grown *E. coli*. Notably, anaerobically grown cells grew faster than those grown in the presence of oxygen, even at the highest Zn^2+^ concentration, although they never reached the same max OD_600_ levels as aerobically grown cells. With a moderate amount of added Zn^2+^ (0.5 mM), *E. coli* cells grown aerobically with added Zn^2+^ acquired >4-fold more zinc than when grown in LB with no added Zn^2+^ whereas anaerobically grown cells did not (Fig. 2, Tables S2, and S3). The effects of oxygen on zinc uptake in *E. coli* have not been previously investigated although a previous study showed that *E. coli* grown aerobically accumulated increased iron and copper with zinc stress (0.1 mM Zn^2+^) (43). Although we did not detect an increase in iron with zinc stress (0.5 mM Zn^2+^) here, the previous work used a minimal medium for the zinc stress analyses rather than LB as reported here. A limitation of using ICP-MS for quantifying cellular metal content, however, is that it cannot discriminate between the labile and tightly bound metal pools and it cannot measure dynamic metal uptake in living cells.

Fluorescent protein-based Zn^2+^ sensors were used to gain further insight to the effects of oxygen on zinc homeostasis in living cells. After confirming reversible and reproducible responses from CreiLOV_N41C_ in aerobic and anaerobic *E. coli* MG1655 (Figs. 3*A* and 4) and ZapCY1 and ZapCY2 in aerobic *E. coli* (Fig. 3, *B* and *C*), sensors were applied to monitor changes in endogenous zinc levels with added zinc. Two approaches were taken. In the first approach, cells were grown, and protein was expressed in the presence of added Zn^2+^, then cells were harvested and transferred to metal-free medium for evaluation. In the second approach, cells were grown, and protein was expressed with no added Zn^2+^ prior to harvesting cells and then Zn^2+^ was added to cell suspensions in metal-free medium during the experiment. In the former approach, we found that the growth and protein expression conditions impacted the results. Many previous applications of fluorescent protein-based sensors have focused on mammalian cells rather than bacteria, which have much smaller cell sizes that necessitate careful control of protein expression to both avoid aggregation and maintain sufficient brightness and sensitivity for metal detection. When protein was quickly overexpressed (37 °C, 3 h), inclusion bodies could form in the cells and lead to inconsistent sensor response and high error bars (Figs. 5 and S6). Under these conditions, endogenous Zn^2+^ was detected only when cells were grown with 2 mM ZnCl_2_ added to LB medium, although with high error. More consistent results were obtained under lower temperature expression conditions (18 °C, overnight), where endogenous zinc was no longer detected even with 2 mM added ZnCl_2_. Both the more consistent sensor response and longer expression time, which could allow the cells time to respond to the added Zn^2+^, likely led to the lack of a sensor response. For comparison, we applied the zinc sensors ZapCY2 and ZapCY1. Only ZapCY1, which has significantly stronger affinity than CreiLOV_N41C_ and ZapCY2, detected ∼3 pM endogenous Zn^2+^ when cells were grown with 2 mM ZnCl_2_. The ZapCY sensors can only be applied under aerobic conditions, however, so we also evaluated zinc uptake in real-time under aerobic and anaerobic conditions using CreiLOV_N41C_ by adding Zn^2+^ directly to the cell suspensions in a 96-well plate during fluorescence monitoring. In these experiments, there were clear differences in the extent of CreiLOV_N41C_ fluorescence quenching by Zn^2+^ between aerobic and anaerobic conditions. Higher concentrations of Zn^2+^ were required to fully quench CreiLOV_N41C_, suggesting that anaerobically grown cells take up less Zn^2+^. Intriguingly, even with no added Zn^2+^, aerobic cells appeared to be scavenging Zn^2+^ from the buffer, as evidenced by moderate and reversible quenching over the time of analysis (∼2 h). On the other hand, CreiLOV_N41C_ in the anaerobic cells was dequenching over the first hour (possibly indicating Zn^2+^ ion efflux), before reequilibrating to the initial fluorescence level over the second hour.

Collectively, these results suggest that oxygen levels affect zinc homeostasis in *E. coli*. Future work will be needed to understand how the molecular responses of anaerobically grown *E. coli* to varied zinc differ from aerobically grown *E. coli*, in terms of both homeostasis mechanisms (*e.g.* regulators and transporters) and zinc utilization in the cells. *E. coli* is well-known to adapt to various external conditions, including the transition from aerobic to anaerobic respiration, by modulating the expression pattern of its genome (44, 45, 46, 47, 48). Although iron and copper homeostasis are known to be impacted by oxygen during the growth of *E. coli*, the effects of aerobic *versus* anaerobic respiration on zinc homeostasis have not been reported (21, 22, 23, 24, 25).

Overall, this work establishes CreiLOV_N41C_ as an oxygen-independent zinc sensor that can be used alongside other protein-based zinc sensors and complementary methods for studying zinc homeostasis in bacteria. Here, we found that *E. coli* grown aerobically and anaerobically have distinct responses to varied zinc. These results have important implications for understanding the effects of metal toxicity in different environments, particularly in host organisms where bacteria are exposed to variations in acidity and oxygen levels. Furthermore, by using complementary methods and sensors, as well as comparing the effects of protein expression conditions on sensor performance in *E. coli*, this work lays a foundation for studying zinc homeostasis in other anaerobic bacteria using CreiLOV_N41C_.

## Experimental procedures

### General information

All reagents were purchased from commercial sources and used as received. Primers were purchased from Sigma-Aldrich. *E. coli* strain MG1655 was from American Type Culture Collection and made chemically competent by CaCl_2_. *E. coli* strain DH5α for cloning was obtained from New England Biolabs. Restriction enzymes, T4 DNA ligase, Q5 DNA polymerase, the Gibson assembly kit, and PCR cleanup kit were from New England Biolabs. Plasmid DNA miniprep kit was from Zymo Research. Fluorescence spectra were obtained on an Agilent Eclipse spectrofluorometer using quartz cuvettes (Starna cells) with 1 cm path lengths or on a Tecan Spark plate reader using 96-well black flat-bottom plates (Greiner Bio-One). All measurements were conducted at 25 °C and maintained by a circulating water bath. To remove metal ions, HEPES buffer was treated with Chelex-100 resin (Bio-Rad) according to the manufacturer’s batch protocol. Fluorescence data were obtained by exciting at 450 nm for CreiLOV_N41C_ and 433 nm for ZapCY1 and ZapCY2. For CreiLOV_N41C_, excitation and emission slit widths were set to 10 nm, and emission acquired from 470 to 570 nm and from 475 to 580 nm on the spectrofluorometer and plate reader, respectively. For ZapCY1 and ZapCY2, excitation and emission slit widths were set to 5 and 10 nm on spectrofluorometer and plate reader, respectively. The emission was acquired from 443 to 570 nm and from 458 to 580 nm on spectrofluorometer and plate reader, respectively. Growth curve experiments were monitored using Tecan Spark plate reader with 96-well clear round-bottom plates (Greiner Bio-One). Experiments involving CreiLOV_N41C_ were done anaerobically using buffer that was freshly degassed with argon. Anaerobic manipulations were done in an anaerobic chamber, using cuvettes with screw caps and septa and a gas-tight Hamilton syringe, adding mineral oil on top of the culture and using gas-sealed tape around the 96-well plate, or using a BD GasPak EZ anaerobe pouch and a gas generating sachet with anaerobic indicator. We note that higher error bars observed for some of the anaerobic measurements likely arise from the additional time required for reagent additions compared to aerobic conditions.

### Growth curves of *E. coli* in the presence of varied concentrations of Zn^2+^

Single colonies of *E. coli* MG1655 were selected and grown in LB medium overnight at 37 °C and 250 rpm. The starter culture was inoculated at 1/100 dilution into fresh LB medium with either ZnCl_2_ or zinc gluconate (0–2 mM) and grown in a 96-well plate for 65 h at 37 °C with shaking. For anaerobic conditions, the starter culture was inoculated at 1/100 dilution into LB medium containing glucose (20 mM) as a carbon source, KNO_3_ (20 mM) as an electron source, and either ZnCl_2_ or zinc gluconate (0–2 mM) and grown anaerobically for 65 h at 37 °C. For both conditions, the OD_600_ was recorded every 30 min on a Tecan Spark plate reader. The growth parameters (lag time, growth rate, and max OD_600_) were calculated using nonlinear regression curve fitting to a four-parameter logistic equation with GraphPad Prism 9 software (GraphPad software, CA; https://www.graphpad.com) (49, 50, 51). Anaerobic manipulations were done in an anaerobic chamber or by adding mineral oil on top of the culture and using gas-sealed tape around the 96-well plates.

### ICP-OES analysis of metals in the growth media

LB (2 ml) and LB supplemented with 0.5 mM ZnCl_2_ (2 ml) media were boiled in metal-free plastic centrifuge tubes (Eppendorf) until the media were dry. Dried media were digested with 560 μl Milli-Q water and 60 μl trace metal concentrated HNO_3_ at 80 °C overnight. Digested media were diluted with Milli-Q water to bring to a total volume of 2.5 ml. The samples were analyzed by an Agilent 725 Simultaneous ICP-OES equipped with VistaChip II charge coupled device detector and image mapping technology to provide complete wavelength coverage from 167 to 785 nm. Trace metal HNO_3_ (2%) was used as the blank. Metal concentrations in media were measured by ICP-OES for Ca, Cr, Cu, Fe, K, Mg, Mn, Na, Ni, Pb, Se, V, Zn, and Mo. ICP-OES calibration standards contained 0.2, 0.5, 1.0, 2.0, and 5.0 ppm for each metal and were all prepared by diluting commercially available Inorganic Ventures' ICP-MS Complete Standard 71A (10 ppm) and Inorganic Ventures' ICP-MS Refractory Elements Standard 71B (10 ppm) with 2% trace metal HNO_3_.

### ICP-MS analysis of *E. coli* cells

The starter culture was inoculated at 1/100 dilution into fresh LB medium supplemented with ZnCl_2_ (0 or 0.5 mM) and grown at 37 °C and 250 rpm until OD_600_ reached 0.6 to 0.8. For anaerobic conditions, the starter culture was inoculated at 1/100 dilution into LB medium containing glucose (20 mM) as a carbon source, KNO_3_ (20 mM) as an electron source, and ZnCl_2_ (0 or 0.5 mM) and grown anaerobically at 37 °C until OD_600_ reached 0.6 to 0.8. Immediately after growth and prior to harvesting cells, live culture from each sample was collected to perform serial dilution from 10^−1^ to 10^−5^ in 0.85% saline solution for determining the colony-forming unit (CFU) count. For each dilution from 10^−3^ to 10^−5^, 10 μl of diluted culture was plated on an LB agar plate and incubated at 37 °C overnight. CFU/ml were both manually counted and automatically counted by ImageJ software. Cell pellets were harvested (4 °C, 5000*g*) and washed with Milli-Q water, 1 mM EDTA (3x), and then Milli-Q water. Cell pellets were dried overnight at 80 °C, weighed, and digested with 100 μl trace metal concentrated HNO_3_ at 65 °C for 30 min, then increased to 100 °C for 5 h. Digested cells were diluted with 1.4 ml Milli-Q water. All centrifuge tubes, containers, Teflon tubes, and pipet tips are plastic and were acid-washed before use. Cell samples were analyzed with an Agilent 8800 triple-quadrupole ICP-MS instrument (Agilent Technologies) equipped with an SPS 4 autosampler and using 2% trace metal HNO_3_ as the blank. The following settings were fixed for analysis: cell entrance, −50 V; cell exit, −70 V; plate bias, −70 V; octP bias, −18 V; collision cell helium flow, 4.3 ml/min. Optimal voltages for Extract 2, Omega Bias, Omega Lens, OctP RF, and Deflect were determined *via* auto tune with 1 ppb instrument tuning solution before each sample set was analyzed. Samples were introduced by a peristaltic pump with 0.5-mm-internal-diameter tubing through a MicroMist borosilicate glass nebulizer (Agilent Technologies). Samples were initially taken up at 0.3 rps for 60 s then stabilized for 15 s at 0.1 rps. Samples were analyzed in spectrum mode at 0.1 rps, and three replicates of 100 sweeps were performed for each element analyzed. Sampling probe and tubing were rinsed for 90 s at 0.3 rps with 2% trace metal HNO_3_ after every sample. Agilent MassHunter Workstation (https://www.agilent.com/en/product/software-informatics/mass-spectrometry-software/data-acquisition) was used for data acquisition and analysis. ICP-MS calibration standards were prepared in the same manner as ICP-OES standards, and contained 0.5, 1.0, 2.5, 5, 10, 16, 25, 40, 50, 100, 200, 500, 750, and 1000 ppb for each metal. Metal content was quantified by converting metal concentrations in ppb to atoms/CFU using CFU values calculated from serial dilutions corresponding to each sample (described above). Metal contents of cells were measured by ICP-MS for Na, Mg, K, Ca, V, Cr, Mn, Fe, Ni, Cu, Zn, As, Se, Mo, and Pb.

### Molecular cloning

The plasmid pQE80L-CreiLOV_N41C_ was reported previously (31). The plasmid pUC57-Zap1 was from GenScript. The plasmid pBAD-ZapCY2 (unpublished) was previously prepared using pcDNA3-ZapCY2 (Addgene #36320) and pBAD-His-miRFP709 (Addgene #79986) (34, 52). All plasmids were verified by DNA sequencing.

#### pQE80L-ZapCY2

ZapCY2 was PCR amplified from pBAD-ZapCY2 using the primers listed below. The pQE80L vector from pQE80L-CreiLOV_N41C_ was digested with BamHI and HindIII restriction enzymes. ZapCY2 was inserted between BamHI and HindIII restriction sites in the pQE80L vector. The primers are as follows: sense: 5′–CAGATCTGAGCTCGGATCC–3′ and antisense: 5′–GCCAAAACAGCCAAGCTTCG–3′

#### pQE80L-ZapCY1

Zap1 from pUC57-Zap1 and the pQE80L vector from pQE80L-ZapCY2 were PCR amplified and assembled by Gibson Assembly. Primers for each amplification are listed as follows: Zap1 sense: 5′–CGCCCGCATGCATAAAAACAATGACTTAAAATGCAAATGG–3′; Zap1 antisense: 5′–CTCACCATGAGCTCGATACCATGTTGACAATTAATATGG–3′; pQE80L sense: 5′–TCGAGCTCATGGTGAGCAAGGGCGAG–3′; pQE80L antisense: 5′–TTATGCATGCGGGCGGCGGT–3.

### Live cell suspension assays for detecting intracellular labile Zn^2+^

The pQE80L-CreiLOV_N41C_, pQE80L-ZapCY1, and pQE80L-ZapCY2 plasmids were transformed into *E. coli* MG1655 and grown in LB agar plates containing ampicillin (100 μg/ml). Single colonies were inoculated into LB medium containing ampicillin (100 μg/ml) and grown overnight at 37 °C and 250 rpm. Starter cultures were inoculated at 1/100 dilution into fresh LB media containing ampicillin (100 μg/ml) and incubated at 37 °C with shaking until the cultures reached OD_600_ of 0.3 to 0.6. Protein expression was then induced with IPTG (0.1 mM), and cells were incubated overnight at 18 °C. Bacterial cells were harvested by centrifugation (4500 rpm, 4 °C, 10 min) and washed twice with metal-free HEPES buffer (50 mM HEPES and 100 mM NaCl at pH 7). The cells were resuspended in fresh HEPES buffer to a final OD_600_ of ∼1 for CreiLOV_N41C_ samples and ∼0.5 for ZapCY1 and ZapCY2 samples. The cell suspension (3 ml) was transferred to a clean quartz cuvette. The initial fluorescence was recorded, followed by the fluorescence upon the addition of TPEN. The TPEN concentration was 50 μM for CreiLOV_N41C_ samples and 5 μM for ZapCY1 and ZapCY2 samples. ZnCl_2_, saponin, and pyrithione were then added, and the fluorescence was recorded. ZnCl_2_/saponin/pyrithione concentrations were 100 μM/0.002%/1.5 μM for CreiLOV_N41C_ samples and 10 μM/0.002%/1.5 μM for ZapCY1 and ZapCY2 samples. Lastly, the fluorescence was recorded after the addition of TPEN. TPEN concentrations were 200 μM for CreiLOV_N41C_ samples and 20 μM for ZapCY1 and ZapCY2 samples. We note that although 50 μM TPEN and 100 μM ZnCl_2_ were initially used to treat cells with ZapCY series sensors, the ZapCY1 response to zinc was inconsistent under these conditions. ZapCY1 and ZapCY2 were ultimately evaluated using concentrations of 5 μM for TPEN and 10 μM ZnCl_2_ because the response of ZapCY1 to zinc was consistent under these treatment conditions.

### Fluorescence microscopy imaging of live *E. coli* cells expressing CreiLOV_N41C_

*E. coli* MG1655 cells transformed with pQE80L-CreiLOV_N41C_ were grown, and protein expression was induced as described for the live cell suspension assays for detecting intracellular labile Zn^2+^. Cell pellets were then harvested, washed, and resuspended in metal-free HEPES buffer as described above. Cell suspension (1 μl) was pipetted onto a thin layer of 3% agarose on a general microscope slide and the cover glass was placed on top. For anaerobic conditions, the starter cultures were inoculated at 1/100 dilution into LB medium containing glucose (20 mM) as a carbon source, KNO_3_ (20 mM) as an electron source, and ampicillin (100 μg/ml). The cultures were grown anaerobically and statically and protein expression was induced as described for the live cell suspension assays for detecting intracellular labile Zn^2+^. Cell pellets were then harvested, washed, and resuspended in metal-free HEPES buffer as described above. A BD GasPak EZ anaerobe pouch was prepared with a 3.5 × 1.5 cm hole on one of the faces of the pouch. The hole was covered by a cover glass and sealed with epoxy as previously described (42). The microscope slide with the sample was placed inside the pouch on the bottom of the cover glass. A gas generating sachet with anaerobic indicator was placed inside the pouch to keep the slide under anaerobic conditions during imaging. Imaging experiments were performed by using an ECHO Revolve microscope equipped with a 5MP CMOS Monochrome Camera (Fluorescence) and 12 MP Color Camera. The light source was a LED light cube-FITC(M). Fluorescence images were obtained with an oil-immersion objective at 100 × magnification. The exposure time, sensitivity, and contrast for acquisition of fluorescence images were kept constant for each series of images for each channel. Images were obtained by exciting at 470/40 nm. Both emission and brightfield images were obtained. The following procedure was carried out for aerobic and anaerobic conditions: images were collected initially and 5 min after the addition of TPEN (50 μM). ZnCl_2_/saponin/pyrithione (100 μM/0.002%/1.5 μM) was added to the cells, and images were recorded 15 min after the addition of ZnCl_2_. Lastly, TPEN (200 μM) was added to the cells and images were recorded 20 min after the treatment of TPEN. Anaerobic manipulations were done in an anaerobic chamber or using a BD GasPak EZ anaerobe pouch and a gas generating sachet with anaerobic indicator, and epoxy. Images were analyzed with ImageJ (http://imagej.nih.gov/ij/). Using an intensity threshold, each bacterial cell was selected, and the mean intensity measured by ImageJ. The background intensity was measured by selecting regions of the image without any cells and subtracted from the cellular intensities. The mean intensity per cell was used to calculate the sensor intensity initially, with TPEN, with Zn^2+^/saponin/pyrithione, and with subsequent TPEN.

### Live cell suspension assays for detecting endogenous Zn^2+^ in *E. coli* grown with varied zinc concentrations

Starter cultures were inoculated at 1/100 dilution into fresh LB medium with either ZnCl_2_ or zinc gluconate (0–1.5 mM) and ampicillin (100 μg/ml), and starter cultures were inoculated at 1/25 dilution into LB medium containing either ZnCl_2_ or zinc gluconate (2 mM) and ampicillin (100 μg/ml). Cultures were grown at 37 °C with shaking to an OD_600_ of 0.3 to 0.6. Protein expression was then induced with IPTG (0.5 mM) for 3 h at 37 °C or with IPTG (0.1 mM) overnight at 18 °C. For anaerobic conditions, starters cultures were inoculated at 1/100 dilution into fresh LB medium supplemented with glucose (20 mM) as carbon source, KNO_3_ (20 mM) as electron source, ZnCl_2_ or zinc gluconate (0–1.5 mM), and ampicillin (100 μg/ml), and starter cultures were inoculated at 1/25 dilution into the LB with the same ingredients and high concentration of ZnCl_2_ or zinc gluconate (2 mM). Cultures were grown in an anaerobic chamber at 37 °C to an OD_600_ of 0.3 to 0.6. Protein expression was induced with IPTG (0.5 mM) for 3 h at 37 °C. The following procedure was carried out for aerobic and anaerobic conditions: bacterial cells were centrifuged at 4500 rpm, 4 °C, and 10 min. The cell pellets were washed twice and resuspended in metal-free HEPES buffer to a final OD_600_ of ∼1 for CreiLOV_N41C_ samples and ∼0.5 for ZapCY1 and ZapCY2 samples. The cell suspension (3 ml) was transferred to a clean quartz cuvette or a cuvette with screw cap and septum inside an anaerobic chamber. The initial fluorescence was recorded, followed by the fluorescence upon the treatment of TPEN (50 μM) and after addition of ZnCl_2_/saponin/pyrithione (100 μM/0.002%/1.5 μM). Anaerobic manipulations were performed in anaerobic chamber or using cuvettes with screw caps and septa and a gas-tight Hamilton syringe.

### Sensing zinc influx in *E. coli* using CreiLOV_N41C_ and ZapCY2

*E. coli* MG1655 cells transformed with pQE80L-CreiLOV_N41C_ and pQE80L-ZapCY2 were grown aerobically and, in the case of CreiLOV_N41C_ also anaerobically, and protein expression was induced as described for the live cell suspension assays for detecting intracellular labile Zn^2+^. Cell pellets were then harvested, washed, and resuspended in metal-free HEPES buffer as described above. The cell suspension (200 μl) was transferred to a 96-well plate. The initial fluorescence was recorded, followed by the fluorescence upon the addition of ZnCl_2_ (0–75 μM). Fluorescence was recorded every 10 min up to 1.5 h for aerobic conditions or up to 2.5 h for anaerobic conditions. TPEN (100 μM for CreiLOV_N41C_ and 150 μM for ZapCY2) was added, and the fluorescence was recorded every 10 min up to 1.5 h for aerobic conditions or up to 2.5 h for anaerobic conditions. Lastly, ZnCl_2_/saponin/pyrithione (200 μM/0.002%/1.5 μM) were added, and the fluorescence was recorded every 10 min up to 1.5 h for aerobic conditions or up to 2.5 h for anaerobic conditions. Anaerobic manipulations were done in an anaerobic chamber or using BD GasPak EZ anaerobe pouch and a gas generating sachet with anaerobic indicator.

## Data availability

All data are contained within the manuscript and in the supporting information.

## Supporting information

This article contains supporting information.

## Conflict of interest

The authors declare that they have no conflicts of interest with the contents of this article.
