# Supplementary material for: Fluorescent protein-based Zn2+ sensors reveal distinct responses of aerobic and anaerobic Escherichia coli cultures to excess Zn2+
Source: J Biol Chem. 2024 Sep 30;300(11):107840. doi: 10.1016/j.jbc.2024.107840 (PMC11550654; doi:10.1016/j.jbc.2024.107840)
Supplement: Supporting information [file mmc1.docx]

**Supplementary materials**

**Fluorescent protein-based Zn^2+^ sensors reveal distinct responses of aerobic and anaerobic *Escherichia* *coli* cultures to excess Zn^2+^**

Hazel N. Nguyen, Uyen Huynh, and Melissa L. Zastrow*

Department of Chemistry, University of Houston, Houston, Texas, 77204, United States

*mzastrow@central.uh.edu

**Table of contents**

| Table S1. Total metal contents in LB and LB supplemented with 0.5 mM ZnCl_2_ | 2 |
| --- | --- |
| Table S2. Total metal contents of *E. coli* grown aerobically in LB and LB supplemented with 0.5 mM ZnCl_2_ | 2 |
| Table S3. Total metal contents of *E. coli* grown anaerobically in LB and LB supplemented with 0.5 mM ZnCl_2_ | 3 |
| Table S4. Total metal contents in HEPES buffer treated with Chelex-100 | 3 |
| Figure S1. Growth kinetics curves for *E. coli* grown aerobically and anaerobically in the presence of varied concentrations of ZnCl_2_ and zinc gluconate | 4 |
| Figure S2. Effects of varied zinc gluconate concentrations on growth kinetics parameters for *E. coli* grown under aerobic and anaerobic conditions | 5 |
| Figure S3. Effects of varied ZnSO_4_ concentrations on the growth of *E. coli* under aerobic conditions | 6 |
| Figure S4. Metal contents in LB and LB containing 0.5 mM ZnCl_2_ | 7 |
| Figure S5. Metal contents of *E. coli* grown aerobically and anaerobically in LB and LB containing 0.5 mM ZnCl_2_ | 8 |
| Figure S6. Microscopy imaging of live *E. coli* cells grown aerobically and expressing CreiLOV_N41C_ | 9 |
| Figure S7. Intracellular zinc in aerobic live *E. coli* cells detected by CreiLOV_N41C,_ ZapCY1, and ZapCY2 | 10 |
| Figure S8. Endogenous zinc as detected by CreiLOV_N41C_ in live cell suspensions of *E. coli* grown aerobically and anaerobically with varied zinc gluconate concentrations | 11 |

**Table S1.** Total metal contents in LB and LB supplemented with 0.5 mM ZnCl_2_.

|  | LB (µM)^a^ | LB + 0.5 mM ZnCl_2_ (µM)^a^ |
| --- | --- | --- |
| Ca | 78 ± 1 | 77 ± 5 |
| Cr | 5.62 ± 0.04 | 5.57 ± 0.01 |
| Cu | 4.38 ± 0.02 | 4.37 ± 0.03 |
| Fe | 7.7 ± 0.1 | 5.28 ± 0.05 |
| K | 3500 ± 50 | 3316 ± 36 |
| Mg | 190 ± 3 | 184 ± 2 |
| Mn | 5.42 ± 0.04 | 5.4 ± 0.1 |
| Na | 72000 ± 870 | 70030 ± 670 |
| Ni | 4.72 ± 0.05 | 4.70 ± 0.04 |
| Pb | 1.39 ± 0.02 | 1.44 ± 0.02 |
| Se | 3.6 ± 0.2 | 3.6 ± 0.3 |
| V | 6.2 ± 0.1 | 6.13 ± 0.02 |
| Zn | 10.5 ± 0.1 | 494 ± 14 |

a. Mo was not detected

**Table S2.** Total metal contents of *E. coli* grown aerobically in LB and LB supplemented with 0.5 mM ZnCl_2_.

|  | LB | | LB + 0.5 mM ZnCl_2_ | |
| --- | --- | --- | --- | --- |
|  | atoms/CFU | nmol/CFU | atoms/CFU | nmol/CFU |
| Na | 2.7(±2.0) × 10^8^ | 4.6(±3.3) × 10^-7^ | 1.5(±0.7) × 10^8^ | 2.4(±1.2) × 10^-7^ |
| Mg | 1.2(±0.8) × 10^8^ | 1.9(±1.3) × 10^-7^ | 6.5(±2.0) × 10^7^ | 1.1(±0.3) × 10^-7^ |
| K | 4.6(±2.5) × 10^7^ | 7.6(±4.2) × 10^-8^ | 3.2(±0.6) × 10^7^ | 5.3(±1.0) × 10^-8^ |
| Ca | 8.1(±2.8) × 10^5^ | 1.3(±0.5) × 10^-9^ | 6.3(±3.9) × 10^5^ | 1.1(±0.7) × 10^-9^ |
| V | 7.4(±5.5) × 10^2^ | 1.2(±0.9) × 10^-12^ | 1.5(±0.3) × 10^3^ | 2.6(±0.5) × 10^-12^ |
| Cr | 3.8(±1.9) × 10^3^ | 6.2(±3.1) × 10^-12^ | 4.2(±2.5) × 10^3^ | 7.0(±4.1) × 10^-12^ |
| Mn | 5.4(±1.5) × 10^4^ | 9.0(±2.4) × 10^-11^ | 4.2(±1.3) × 10^4^ | 7.0(±2.1) × 10^-11^ |
| Fe | 1.6(±0.5) × 10^6^ | 2.7(±0.9) × 10^-9^ | 1.3(±0.3) × 10^6^ | 2.1(±0.5) × 10^-9^ |
| Ni | 2.1(±1.3) × 10^3^ | 3.6(±2.2) × 10^-12^ | 1.7(±0.5) × 10^3^ | 2.8(±0.8) × 10^-12^ |
| Cu | 5.1(±2.4) × 10^4^ | 8.5(±4.0) × 10^-11^ | 6.6(±2.8) × 10^4^ | 1.1(±0.4) × 10^-10^ |
| Zn | 3.2(±1.5) × 10^5^ | 5.3(±2.5) × 10^-10^ | 1.4(±0.7) × 10^6^ | 2.3(±1.3) × 10^-9^ |
| As | 2.4(±0.5) × 10^3^ | 3.9(±0.8) × 10^-12^ | 2.3(±1.1) × 10^3^ | 3.9(±1.8) × 10^-12^ |
| Se | 9.7(±2.1) × 10^3^ | 1.6(±0.3) × 10^-11^ | 8.7(±3.2) × 10^3^ | 1.4(±0.5) × 10^-11^ |
| Mo | 1.1(±0.3) × 10^4^ | 1.9(±0.6) × 10^-11^ | 7.2(±2.4) × 10^3^ | 1.2(±0.4) × 10^-11^ |
| Pb | 8.5(±8.0) × 10^1^ | 1.4(±1.3) × 10^-13^ | 1.9(±1.4) × 10^2^ | 3.2(±2.4) × 10^-13^ |

**Table S3.** Total metal contents of *E. coli* grown anaerobically in LB and LB supplemented with 0.5 mM ZnCl_2_.

|  | LB | | LB + 0.5 mM ZnCl_2_ | |
| --- | --- | --- | --- | --- |
|  | atoms/CFU | nmol/CFU | atoms/CFU | nmol/CFU |
| Na | 3.3(±0.9) × 10^8^ | 5.4(±1.6) × 10^-7^ | 3.5(±0.9) × 10^8^ | 5.8(±1.6) × 10^-7^ |
| Mg | 1.9(±0.3) × 10^8^ | 3.2(±0.5) × 10^-7^ | 1.5(±0.4) × 10^8^ | 2.5(±0.7) × 10^-7^ |
| K | 5.2(±0.7) × 10^7^ | 8.6(±1.2) × 10^-8^ | 2.3(±0.9) × 10^7^ | 3.7(±1.5) × 10^-8^ |
| Ca | 9.3(±2.3) × 10^5^ | 1.5(±0.4) × 10^-9^ | 4.2(±1.7) × 10^5^ | 7.0(±2.9) × 10^-10^ |
| V | 6.9(±2.1) × 10^2^ | 1.2(±0.4) × 10^-12^ | 7.3(±2.7) × 10^2^ | 1.2(±0.5) × 10^-12^ |
| Cr | 9.7(±3.3) × 10^3^ | 1.6(±0.5) × 10^-11^ | 6.8(±2.6) × 10^3^ | 1.1(±0.4) × 10^-11^ |
| Mn | 7.9(±3.3) × 10^3^ | 1.3(±0.6) × 10^-11^ | 2.5(±1.9) × 10^3^ | 4.2(±3.1) × 10^-12^ |
| Fe | 3.6(±1.1) × 10^6^ | 5.9(±1.9) × 10^-9^ | 2.3(±0.3) × 10^6^ | 3.9(±0.5) × 10^-9^ |
| Ni | 1.0(±0.6) × 10^4^ | 1.7(±1.0) × 10^-11^ | 3.7(±0.9) × 10^3^ | 6.2(±1.4) × 10^-12^ |
| Cu | 7.9(±2.9) × 10^4^ | 1.3(±0.5) × 10^-10^ | 5.2(±1.8) × 10^4^ | 8.7(±2.9) × 10^-11^ |
| Zn | 4.4(±0.8) × 10^5^ | 7.4(±1.2) × 10^-10^ | 2.5(±0.9) × 10^5^ | 4.2(±1.5) × 10^-10^ |
| As | 1.5(±0.2) × 10^3^ | 2.4(±0.4) × 10^-12^ | 1.2(±0.4) × 10^3^ | 2.0(±0.7) × 10^-12^ |
| Se | 1.7(±0.6) × 10^4^ | 2.8(±0.9) × 10^-11^ | 6.8(±2.5) × 10^3^ | 1.1(±0.4) × 10^-11^ |
| Mo | 4.8(±1.1) × 10^4^ | 7.9(±1.9) × 10^-11^ | 2.6(±0.8) × 10^4^ | 4.3(±1.3) × 10^-11^ |
| Pb | 8.2(±6.1) × 10^1^ | 1.4(±1.0) × 10^-13^ | 6.1(±3.5) × 10^1^ | 1.0(±0.6) × 10^-13^ |

**Table S4.** Total metal contents in HEPES buffer treated with Chelex-100

|  | µM |
| --- | --- |
| Ca | 0.57 ± 0.02 |
| Cr | Not detected |
| Cu | Not detected |
| Fe | 0.010 ± 0.002 |
| K | 14.2 ± 4.3 |
| Mg | 0.030 ± 0.002 |
| Mn | Not detected |
| Ni | 0.18 ± 0.02 |
| Pb | Not detected |
| Se | 0.040 ± 0.006 |
| V | Not detected |
| Zn | 0.170 ± 0.002 |
| Mo | Not detected |


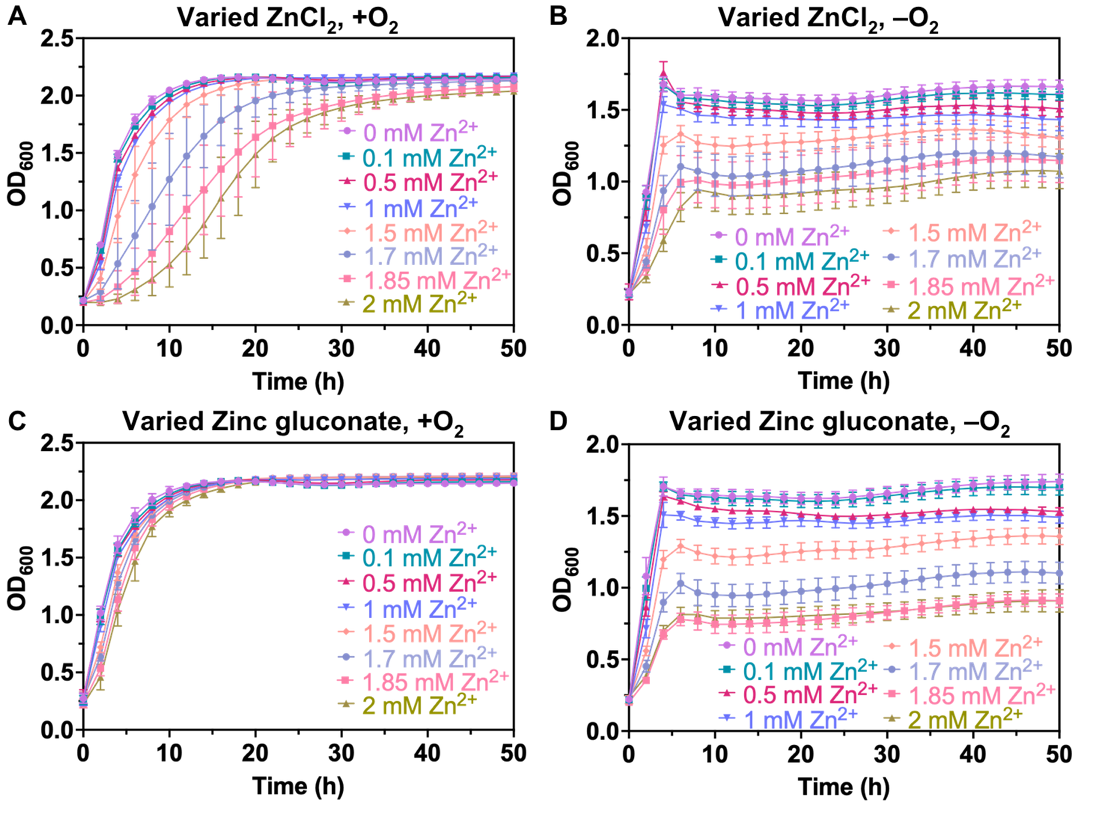


**Figure S1.** Growth kinetics curves for E. coli grown aerobically and anaerobically in the presence of varied concentrations of ZnCl_2_ and zinc gluconate. (A) E. coli grown aerobically with varied ZnCl_2_, (B) E. coli grown anaerobically with varied ZnCl_2_, (C) E. coli grown aerobically with varied zinc gluconate, (D) E. coli grown anaerobically with varied zinc gluconate. Growth curves are shown with error bars as SD from 3 biological replicates.


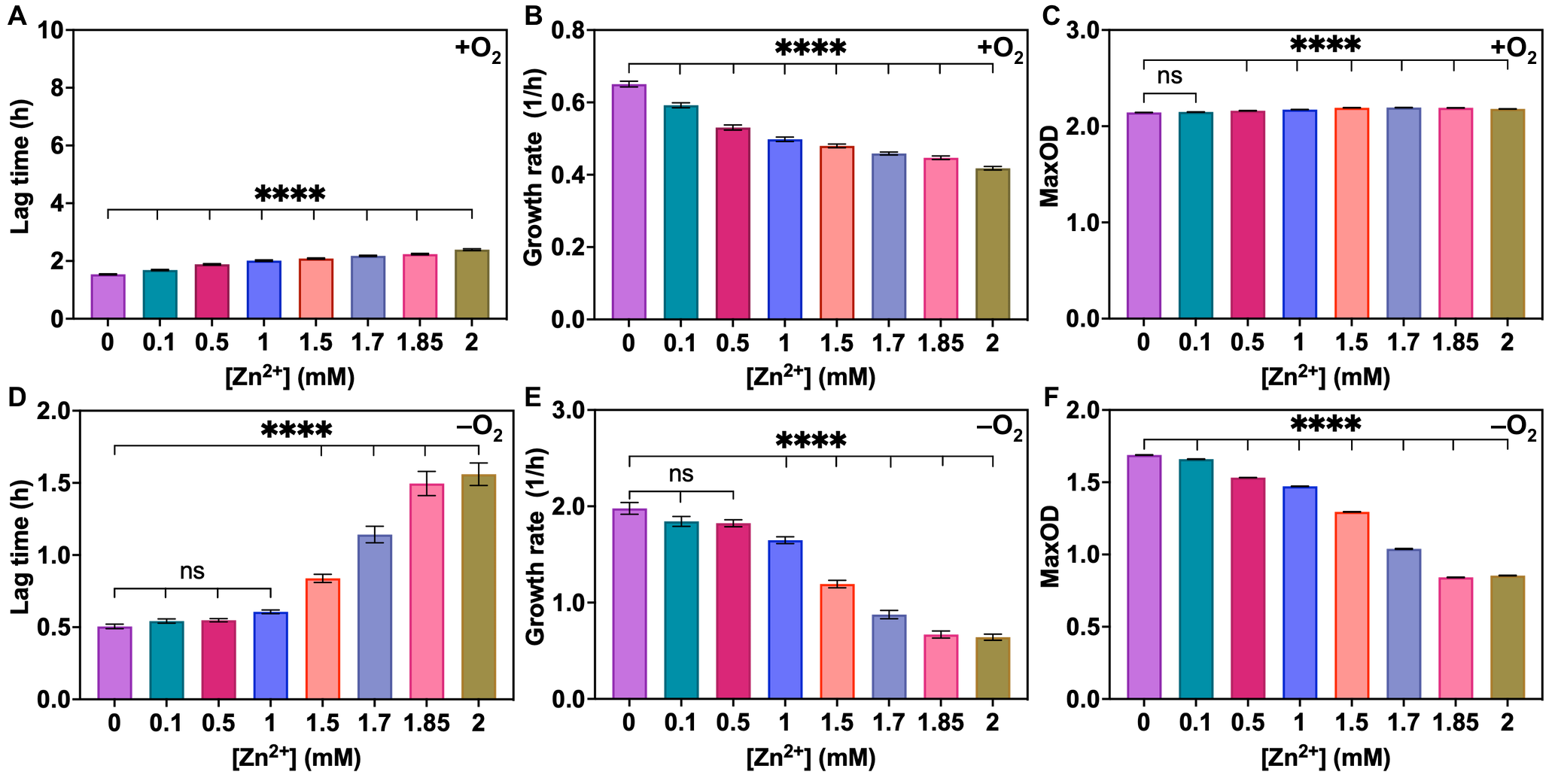


**Figure S2.** Effects of varied zinc gluconate concentrations on growth kinetics parameters for E. coli grown under aerobic (labeled with +O_2_) and anaerobic (labeled with –O_2_) conditions. (A-C) Aerobically grown E. coli, (D-F) Anaerobically grown E. coli. Lag time, growth rate, and max OD_600_ represent the mean ± SEM of 3 biological replicates. Growth parameters were calculated using nonlinear regression curve fitting to a four-parameter Logistic equation with GraphPad Prism 9 software. NS, not significant; ****P ≤ 0.0001 as determined by one-way ANOVA with Tukey multiple comparison test.


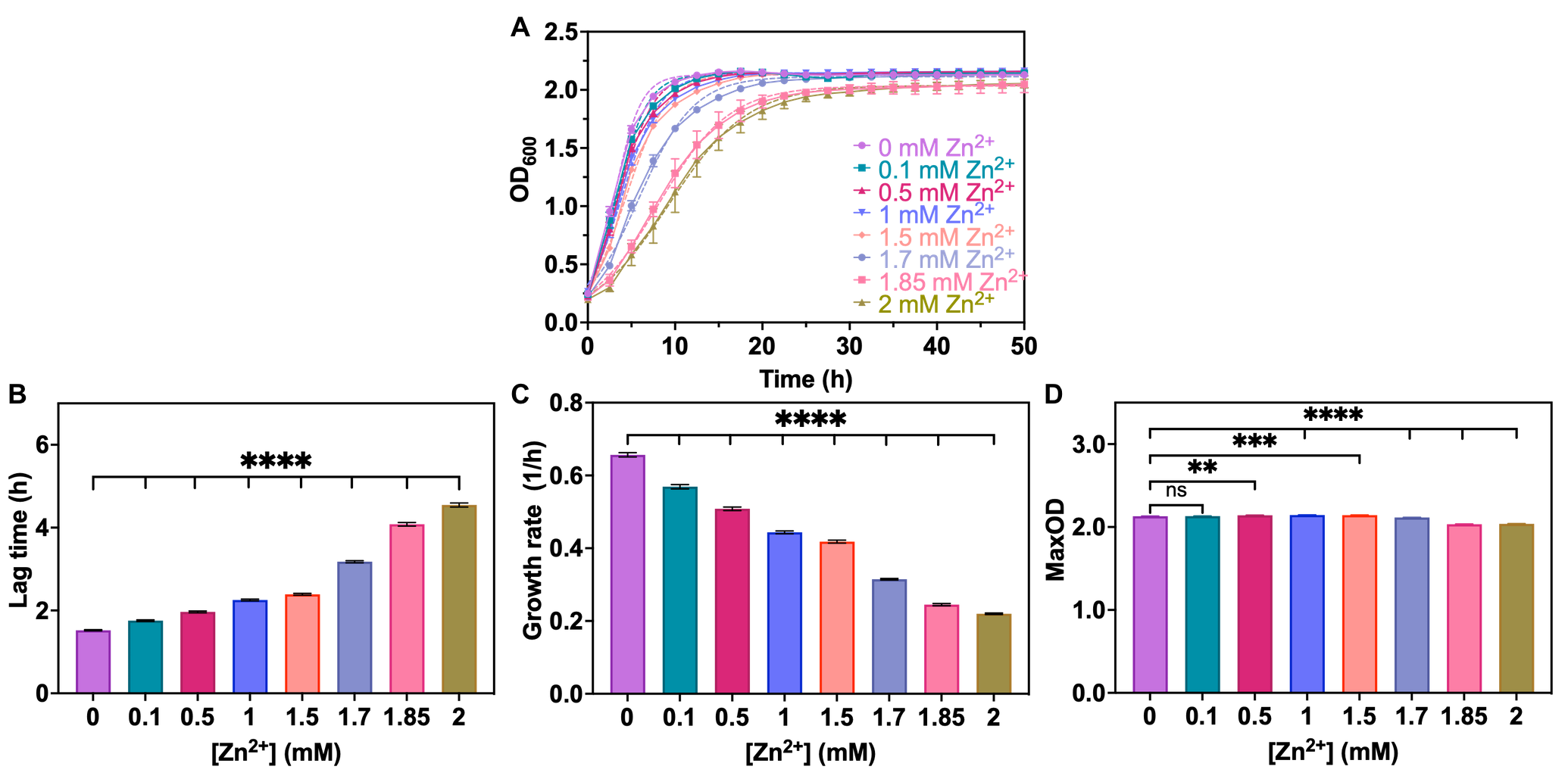


**Figure S3.** Effects of varied ZnSO_4_ concentrations on the growth of E. coli under aerobic conditions. (A) Growth kinetics curves with error bars as SD from 3 biological replicates. (B-D) Lag time, growth rate, and max OD_600_ represent the mean ± SEM of 3 biological replicates. Growth parameters were calculated from the growth curves using nonlinear regression curve fitting to a four-parameter Logistic equation with GraphPad Prism 9 software. NS, not significant; **P ≤ 0.01; ***P ≤ 0.001; ****P ≤ 0.0001 as determined by one-way ANOVA with Tukey multiple comparison test.

**Figure S4.** Quantification of metal contents in LB and LB supplemented with 0.5 mM ZnCl_2_ as determined by ICP-OES. Data are shown as individual data points overlaid on the bar chart representation, with error bars as SD from 3 technical replicates. Data are presented using a log10-scale for the y-axis.

**
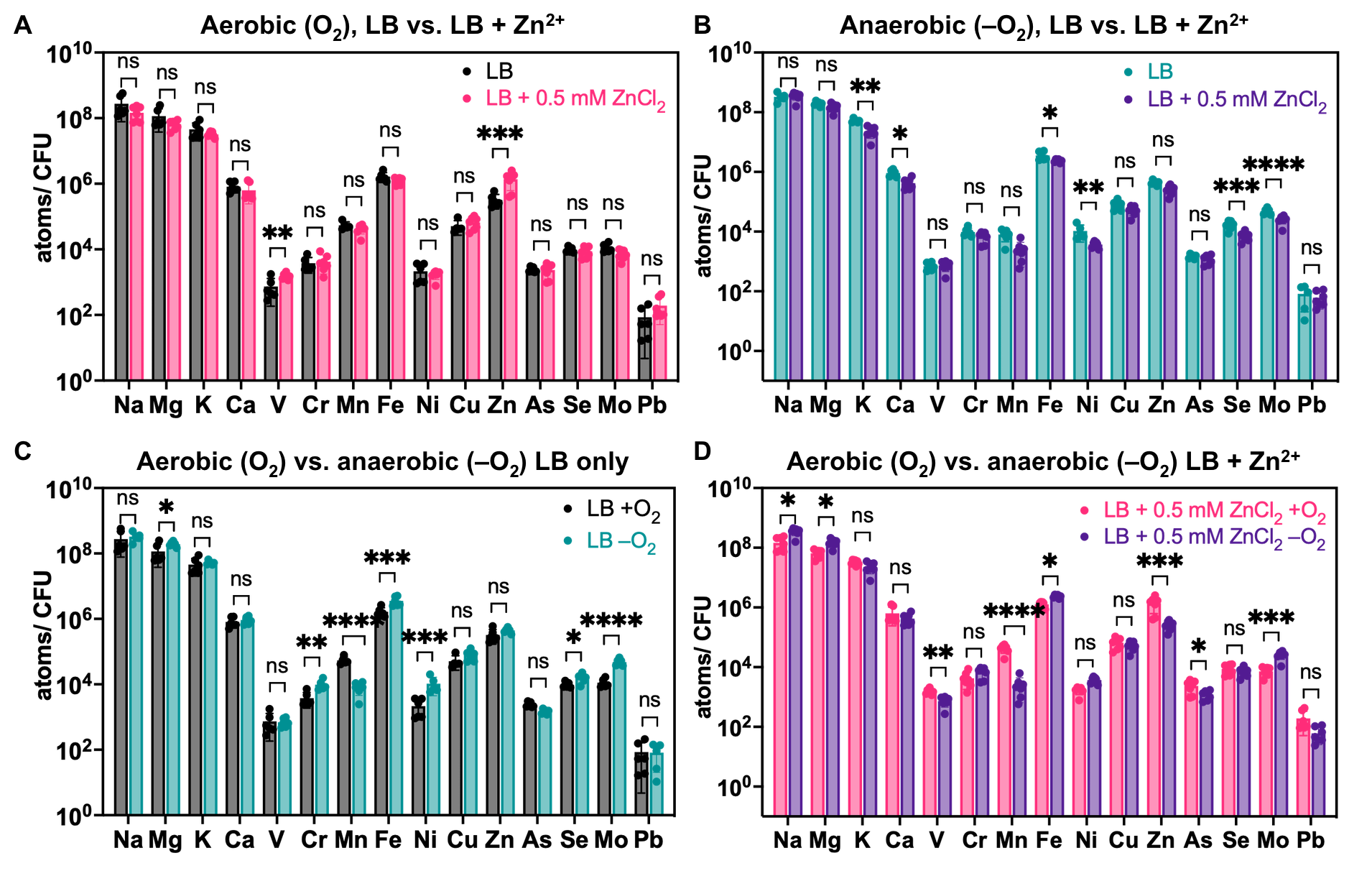
**

**Figure S5.** ICP-MS quantification of metal contents of E. coli grown aerobically and anaerobically in LB and LB containing 0.5 mM ZnCl_2_. Pairwise comparisons of the four separate conditions are shown. (A) E. coli grown aerobically in LB with and without 0.5 mM added ZnCl_2_, (B) E. coli grown anaerobically in LB with and without 0.5 mM added ZnCl_2_, (C) E. coli grown in LB only under aerobic and anaerobic conditions, (D) E. coli grown in LB containing 0.5 mM added ZnCl_2_ under aerobic and anaerobic conditions. Data are shown as individual data points overlaid on the bar chart representation, with error bars as SD from 3 biological replicates. Data are presented using log10-scale for the y-axis. NS, not significant; *P ≤ 0.05; **P ≤ 0.01; ***P ≤ 0.001; ****P ≤ 0.0001 as determined by one-way ANOVA with Tukey multiple comparison test.


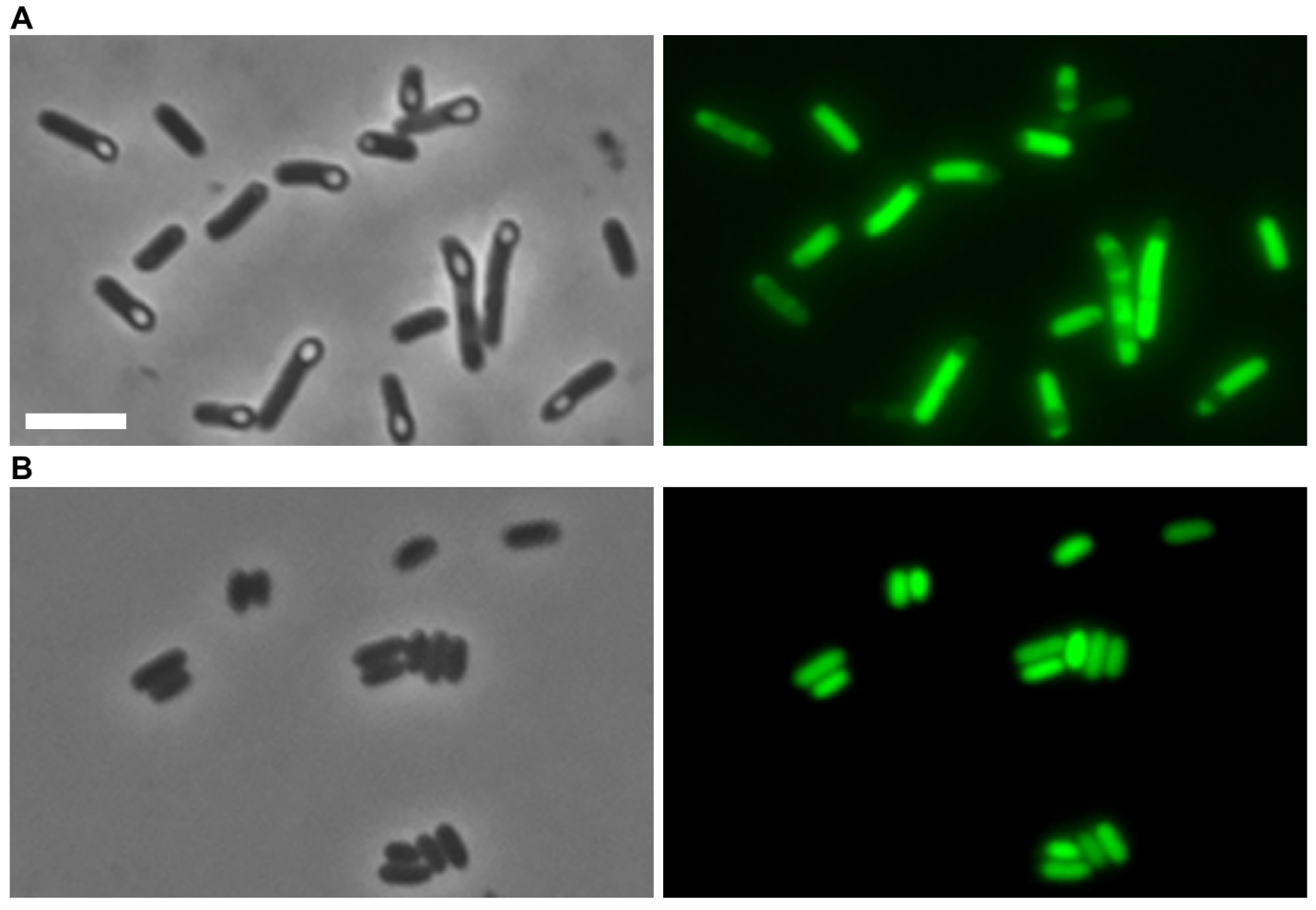
**Figure S6.** Microscopy imaging of live E. coli cells grown aerobically and expressing CreiLOV_N41C_. (A) Brightfield (left) and green channel fluorescence signal (right) of cells grown using initial growth and protein expression conditions (37 °C, 0.5 mM IPTG, 3 h), (B) Brightfield (left) and green channel fluorescence signal (right) of cells grown using optimized protein expression conditions (18 °C, 0.1 mM IPTG, overnight). Scale bar = 5 µm.


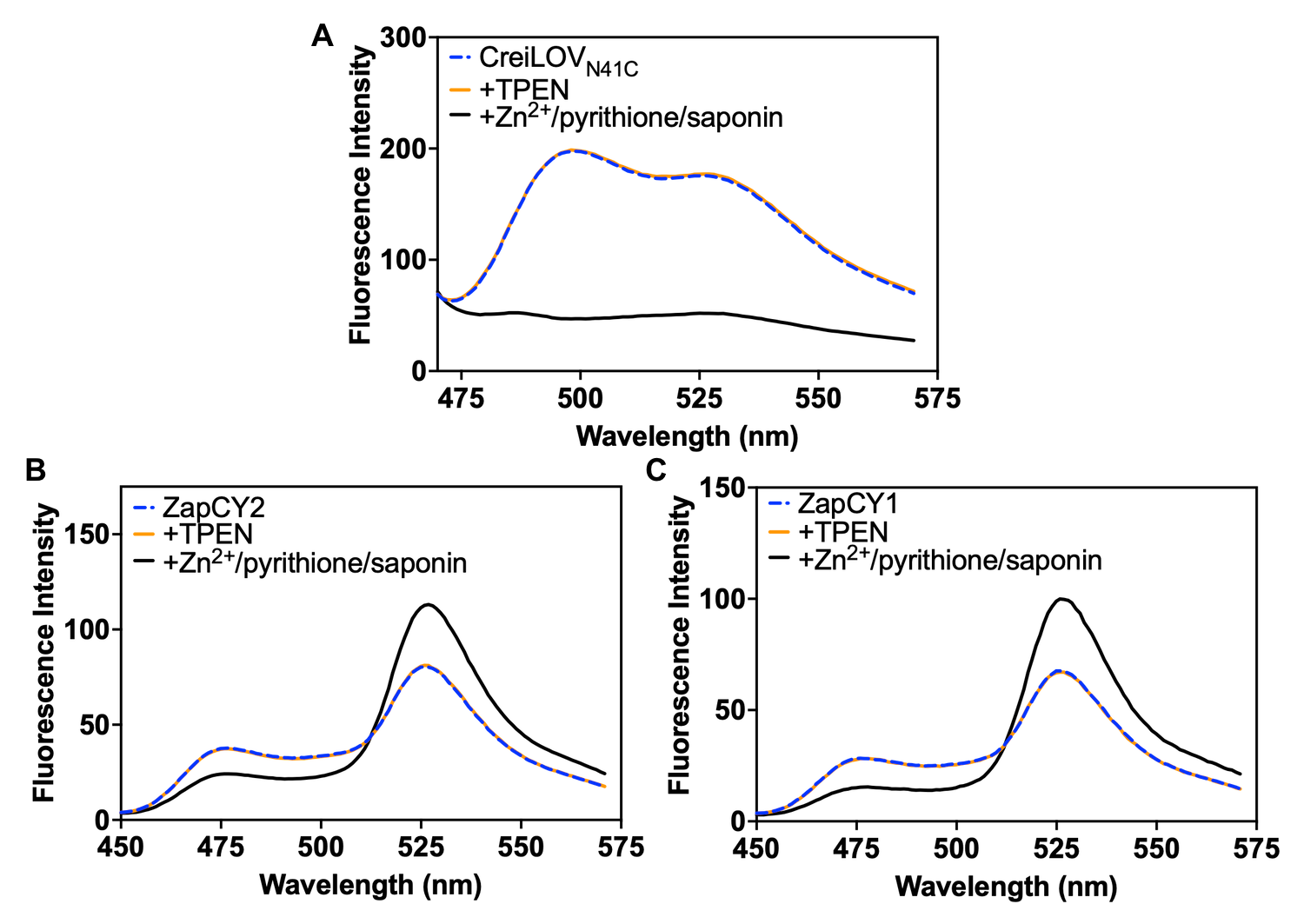


**Figure S7.** Intracellular zinc in aerobic live E. coli cells detected by fluorescent protein based Zn^2+^ sensors. (A) CreiLOV_N41C_, (B) ZapCY2, and (C) ZapCY1. Initial fluorescence spectra were recorded. TPEN spectra were recorded at 2.5 min (CreiLOV_N41C_) and 5 min (ZapCY2 and ZapCY1) after the addition. ZnCl_2_/saponin/pyrithione spectra were recorded at 8 min (CreiLOV_N41C_), 20 min (ZapCY2), and 15 min (ZapCY1) after the addition. Buffer: 50 mM HEPES, 100 mM NaCl, pH 7.1. λ_ex_ = 450 nm (CreiLOV_N41C_) and 433 nm (ZapCY2 and ZapCY1).


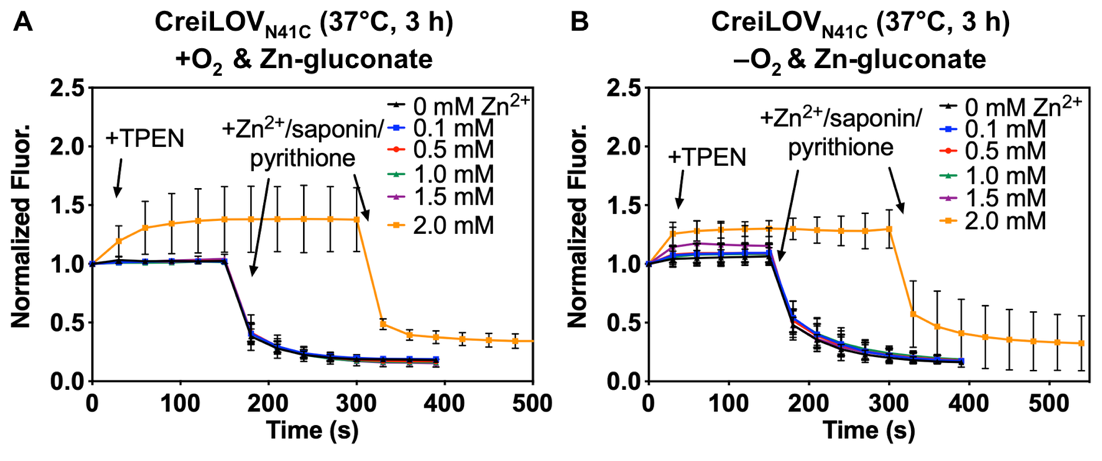


**Figure S8.** Endogenous zinc as detected by CreiLOV_N41C_ in live cell suspensions of E. coli grown (A) aerobically and (B) anaerobically. Protein expression was induced with 0.5 mM IPTG for 3 h at 37 °C and cells were grown in the presence of zinc gluconate (0-2 mM). For each sample, the fluorescence emission was recorded initially and every 30 sec after addition of TPEN (50 µM) and ZnCl_2_/ saponin/ pyrithione (100 µM/ 0.002%/ 1.5 μM). Buffer: 50 mM HEPES, 100 mM NaCl, pH 7.1. λ_ex_ = 450 nm. All error bars represent the standard deviation for 3 or more biological replicates, with ≥2 technical replicates each.
